# Comparing the effectiveness of mother-focused interventions to that of mother-child focused interventions in improving maternal postpartum depression outcomes: A systematic review

**DOI:** 10.1371/journal.pone.0295955

**Published:** 2023-12-20

**Authors:** Divya Kumar, Waqas Hameed, Bilal Iqbal Avan

**Affiliations:** 1 Department of Population Health, Faculty of Epidemiology and Population Health, London School of Hygiene & Tropical Medicine (LSHTM), London, United Kingdom; 2 Department of Community Health Sciences, Aga Khan University Hospital, Karachi, Sindh, Pakistan; Sheffield Hallam University, UNITED KINGDOM

## Abstract

**Background:**

Most empirically researched interventions for postpartum depression (PPD) tend to target mothers’ depression alone. Harmful effects of PPD on physical and mental health of both mother and child has led researchers to investigate the impact of interventions on PPD and child outcomes together. So far, the evidence is limited regarding how these interventions compare with those focusing only on mothers’ depression. This review compares the effectiveness of PPD-improving interventions focusing only on mothers with those focusing on mother and child together.

**Methods:**

Nine electronic databases were searched. Thirty-seven studies evaluating mother-focused (n = 30) and mother-child focused interventions (n = 7) were included. Under each category, three theoretical approaches—psychological, psychosocial and mixed—were compared using standardized qualitative procedures. The review’s primary outcome was maternal PPD.

**Results:**

A higher proportion of mother-focussed interventions [20/30 (66.7%)] brought significant reduction in PPD outcomes as compared to a lower proportion of mother-child focused interventions [4/7 (57.14%)]. Mother-focused mixed approaches [3/3 (100%)] performed better in improving PPD than psychological [16/24 (67%)] or psychosocial approaches [1/3 (33.3%)] alone. Amongst mother-child focused interventions, psychosocial approaches performed well with two-thirds demonstrating positive effects on PPD.

**Conclusion:**

The evidence strongly favors mother-focused interventions for improving PPD with mixed interventions being more effective. Psychosocial approaches performed better with PPD once child-related elements were added, and also seemed best for child outcomes. Psychological approaches were most practiced and effective for PPD, irrespective of the intervention’s focus. Further trials are needed to unpack intervention components that improve PPD and increase uptake, especially in lower-and middle-income countries.

## Introduction

A fairly large volume of evidence has been amassed over the last few decades on different interventions for treating Postpartum Depression (PPD) including pharmacology and psychotherapy [1–4]. Historically, many mothers have been hesitant towards accepting drug treatments to avoid any harmful effects spreading through breast milk [5], thereby, leading to increased non-participation in antidepressant research [6]. This triggered investigation into non-pharmacological interventions for PPD [2, 7]. Over the years, unequivocal findings have been documented regarding the effectiveness of a range of psychological and psychosocial approaches applied by the interventions for treating and preventing PPD in mothers [1, 2, 8].

The fundamental aspect that defines *psychological approaches* has been the formation of a therapeutic alliance between the therapist and their client, maintained by a regular structured verbal exchange between the two; or through a well-structured and defined documented procedure. The clients mostly go through the process themselves with considerable assistance from a therapist or a trained health care professional [2, 9]. Examples include cognitive behaviour therapy (CBT) [2, 10], interpersonal therapy (IPT) [11, 12] and psychodynamic therapy [13, 14]. The *psychosocial approaches* have been defined as those requiring work on one’s social relationships and strengthening social support systems. They have a freer flowing structure and examples include psychoeducation, non-directive counseling, and supportive interactions [8, 15]. Both kinds of approaches require consistent support through phones, at home, online, or at facilities either through individual or group sessions by specialists or non-specialists [2]. CBT based approaches are the most widely researched for PPD [5, 16, 17], although there is still some ambiguity surrounding its effectiveness over other treatments. Having said that, many community-based trials [5, 18–20] have reported significant impact of CBT interventions on depressive symptoms.

PPD, as we know, is a grave public health challenge because of its damaging consequences for the two quite vulnerable groups–women and children. It is defined as a non-psychotic depressive episode occurring within one year of childbirth, though the Diagnostic and Statistical Manual -Version V (DSM-V) classification considers the onset period to begin within 4 weeks while the World Health Organization (WHO) and the Centers for Disease Control (CDC) consider the wider period of one year [4, 21–23]. The prevalence rates for PPD among women residing in High Income Countries (HICs) is approximately 10% and for those in Lower- and middle- income countries (LMIC) is about 20% and more [21, 24, 25], which necessitates generation of public health evidence for varied array of interventions.

Despite plethora of scientific evidence generated over the last 20–25 years, PPD remains an under-diagnosed and under-treated condition [3, 26]. The seriousness of PPD as a disorder stems from the fact that it not only exerts a markedly negative impact on the woman’s mood and her functioning, but also on her interpersonal relationships [27–30] and can further hamper her capacity to provide a nurturing environment to her child [4, 31, 32] leading to detrimental outcomes for the child and the dyadic relationship [7, 29, 33, 34]. Children of mothers with PPD may lack in cognitive, emotional, behavioural and social skills and are at increased risk of developing psychopathology.

Given the impact of PPD on both maternal and child outcomes [35], recent years have seen development of interventions to reduce not only maternal PPD but also strengthen the mother–infant relationship so as to avert any adverse child outcomes [7, 13, 36, 37]. Several systematic reviews of interventions for PPD have pointed that psychological and psychosocial interventions, in fact, positively impact maternal depressed mood irrespective of the school of thought to which they belong [2, 7, 9, 38–40]. However, there is a scarcity of studies that investigate whether there is an impact of an effective PPD treatment on child outcomes [7] or vice versa [41]. Few researchers have tried integrating interventions to investigate their combined impact on maternal PPD as well as child outcomes especially within the context of Early Child Development (ECD) [13, 19, 36, 42]. Unfortunately, the results have been largely inconclusive, one, regarding the key components that bring a positive change in these outcomes and, two, regarding their effectiveness in alleviating PPD specifically.

In the given context, this review attempts to systematically analyze the evidence available on interventions that focus only on mother’s PPD and compare these with interventions that target both maternal PPD and child developmental outcomes in terms of their impact on maternal PPD.

### Objectives/Questions

1. To identify the characteristics including primary approaches of the mother-focused and mother-child focused public health interventions for postpartum depression (PPD).
2. How do the mother-focused interventions compare to mother-child focused interventions in improving maternal PPD outcomes?
3. How do the mother-focused interventions compare to mother-child focused in improving child outcomes?

## Methods

### Database searches

Nine electronic scientific databases were searched: PsycINFO, PubMed, MEDLINE, EMBASE, Global Health, PsycEXTRA, Cochrane Library [(Cochrane Database of Systematic Reviews and Cochrane Central Register of Controlled Trials (CENTRAL)], Scopus and Grey Literature. Search terms used were *postpartum depression (antenatal*, *perinatal*, *postnatal*, *puerperal*, *antepartum*, *postpartum*, *and other synonyms)* AND *Interventions (mother-child*, *maternal and child health*, *psychological*, *psychosocial)*; AND *Intervention studies or evaluation studies including all kinds of trials (randomized controlled trials*, *clinical trials*, *etc*.*)* (see S1 Table A.1 in S1 File for detailed search strategy). Additionally, reference lists of the included articles were hand searched to obtain any relevant papers and studies.

Following the first screening based on titles and abstracts, the full text articles were shortlisted for second screening, during which, data was extracted and recorded in an excel sheet. A data extraction template was designed to record relevant information, such as, location (country), setting (urban/rural), study type and design, baseline assessments, recruitment and retention, description of intervention, outcome measures, type of analysis, and findings. The detailed components of the interventions were extracted from their published articles (n = 38) and related papers or project reports (n = 9). Table 1 below details the inclusion and exclusion criteria for this systematic review.

**Table 1:** Inclusion and exclusion criteria for systematic review.

Criteria	Included	Excluded
**Publication type**	From 01 Jan 2003–01 Dec 2022 English Language Any geographic location	
**Population characteristics**	Women aged ≥18 to ≤50 years Mothers (pregnant women, women who had given birth)	
**Outcome of interest**	Primary outcome Postpartum depression–onset from four weeks to 12 months after birth. This is inclusive of: *(i) PPD scores*: tells us about presence and severity of depressive symptoms/features that may not mean a full PPD diagnosis–assessed by standardized screening tools *(ii) PPD diagnosis*: gives us the status whether a person meets the depression diagnostic criteria or not; assessed by a clinician using standardized diagnostic interview Secondary outcome Child growth Child development	Other concurrent mental disorders, e.g., psychotic disorders, post-partum psychosis, bipolar disorders, substance use disorders, epilepsy and other disabilities Concurrent conditions such as cancer, HIV, cardiovascular disorders and diabetes Child mental health, behavioural problems, pervasive developmental disorders
**Study type**	All intervention studies including Individual and cluster randomized Controlled Trials Controlled before-after studies; controlled interrupted time series studies Clinical trials Non-randomized intervention studies Programs of intervention by government, NGOs or other organizations	Pilot studies, as technically, they may not provide evidence on impact or effectiveness but just a likelihood of the same. They are usually the smaller version of a full-scale study and often referred to as feasibility studies. We wanted to include studies that give us definitive findings related to impact and effectiveness.
**Intervention Approach**	Psychological interventions Psychosocial intervention Mixed interventions (combination of both 1 and 2)	Purely pharmacological interventions for postpartum depression Exercise related therapies including yoga Creative therapies such as art therapies etc.

All the data were analyzed qualitatively. We did not conduct a meta-analysis owing to heterogeneity of data in terms of the components of the intervention, participant characteristics, outcome measures, and varying assessment time points to name a few, which precluded statistical pooling of data.

### Defining the interventions

For this review, the mother-focused interventions were defined as those directed at the mother alone with the primary focus on treating or preventing PPD. On the other hand, we described mother-child focused interventions as those that involved working on enhancing the mother-child interactions and their relationship with the aim of improving both the child health as well as PPD outcomes.

Furthermore, we categorized the two distinctly focused interventions according to three theoretical approaches–psychological, psychosocial and mixed. These categories are well defined in the existing literature and we have used standard terminology to define psychological and psychosocial approaches for the purpose of this review. The rationale for comparing psychological and psychosocial approaches stems from the two having different theoretical underpinnings regarding the process of psychological and behavioural change. The former are mostly referred to as talking therapies and involve sharing one’s thoughts and understanding one’s emotions and behaviours better to be able to bring about a positive behavioural and emotional change. These may also involve discussing one’s personal relationships and/or becoming aware of one’s patterns and defense mechanisms with the aim of improving maladaptive coping [9]. The latter, on the other hand, assume that supportive social relationships have an impact on one’s mental well-being [8, 43] and that emotional (e.g. love, caring, and sympathy) and instrumental support coming from significant others are likely to be the most effective stress buffers [44]. Psychosocial approaches further purport that integration in a social network might directly produce positive psychological states and involves working on developing or enhancing supportive social networks around oneself [8, 15]. These are fundamental differences especially in terms of what they require from both the client/patient/participant and the service provider. Therefore, we felt it was important to consider how each different approach and their various components impacted PPD.

The mixed approaches were defined as those that borrowed elements from both psychological and psychosocial approaches and combined them together, e.g., CBT (psychological) plus non-directive counseling and supportive interactions (both psychosocial).

### Quality of studies

The quality of the studies was assessed using the Scottish Intercollegiate Guidelines Network (SIGN) checklist for Randomized Controlled Trials (RCTs) [45] (see S1 Appendix B in S1 File for Quality Assessment checklist). Based on these criteria, the studies were categorized into: High quality (8–10 score or ++) studies with minimum bias for assessing causality; acceptable quality (5–7 score or +) studies; and low quality (less than 5 score or -) studies having maximum bias as shown in Tables 2 and 3 that describe study characteristics.

**Table 2:** Study characteristics of Mother-focused interventions.

S. No.	Paper	Location	Intervention	Period of intervention	Key maternal outcomes (Other outcomes)	Quality ^a^
Mother-focused: Psychological						
1.	Quasi Experimental **Huynh-Nhu Le (2021)**	USA	CBT	Antepartum + Postpartum	Severity of PPD symptoms + psychopathology	-
2.	RCT **Jannati 2020**	Iran	CBT	Postpartum	Severity of PPD symptoms	+
3.	Cluster RCT **(Ngai 2019)**	Hong Kong	CBT	Antepartum + Postpartum	Severity of PPD symptoms	++
4.	Cluster RCT **Gureje (2019)**	Nigeria	PST	Antepartum + Postpartum	Remission (Child growth + nutrition + development	++
5.	RCT **Lund (2019)**	South Africa	Task-sharing multicomponent	Antepartum + Postpartum	Severity of PPD symptoms + Recovery (Birth outcomes + newborn health + child growth + infant immunization, diarrheal disease and respiratory tract infections)	++
6.	RCT **Fuhr (2019)**	India	BA	Antepartum + Postpartum	Severity of PPD symptoms + Remission	++
7.	Cluster RCT **Sikander (2019)**	Pakistan	BA	Antepartum + Postpartum	Severity of PPD symptoms + Remission	++
8.	RCT **Dimidjian (2017)**	United States of America (USA)	BA	Antepartum	Severity of PPD symptoms	++
9.	RCT **Forsell (2017)**	Sweden	CBT	Antepartum	PPD diagnosis	-
10.	RCT **Pugh (2016)**	Canada	CBT	Postpartum	Severity of PPD symptoms	+
11.	RCT **Milgrom (2016)**	Australia	CBT	Postpartum	Severity of PPD symptoms + PPD diagnosis	+
12.	RCT **Jesse (2015)**	USA	CBT	Antepartum	Severity of PPD symptoms	-
13.	Quasi-experimental study: pre- and post-test design **Ashtiani (2015)**	Iran	CBT	Antepartum	Severity of PPD symptoms (Anxiety symptom severity, religious attitude, Self-esteem)	-
14.	Quasi Experimental **Dimidjian (2015)**	USA	Mindfulness based cognitive therapy (MBCT)	Antepartum	Severity of PPD symptoms + PPD diagnosis (Patient satisfaction)	-
15.	RCT **Tandon (2014)**	USA	CBT	Antepartum + Postpartum	PPD diagnosis	-
16.	RCT **Jiang (2014)**	China	CBT	Postpartum	PPD diagnosis	-
17.	Quasi-experimental study **Hou (2014)**	China	CBT + Systemic Family Therapy	Postpartum	PPD diagnosis (Quality of sleep)	-
18.	RCT **Pinheiro (2013)**	Brazil	CBT and RCT	Postpartum	PPD diagnosis (Anxiety symptoms)	-
19.	RCT **Ammerman (2013)**	USA	CBT	Postpartum	PPD diagnosis + Severity of PPD symptoms	-
20.	RCT **O’Mahen (2013)**	UK	BA	Postpartum	PPD diagnosis (Feasibility, Acceptability)	+
21.	RCT **Milgrom (2011)**	Australia	CBT	Postpartum	Severity of PPD symptoms	-
22.	RCT **Le (2011)**	USA	CBT	Antepartum + Postpartum	Severity of PPD symptoms + PPD diagnosis	-
23.	Cluster RCT **Brugha (2011)**	United Kingdom (UK)	CBT or person centred	Postpartum	Proportion of women with PPD diagnosis	++
24.	RCT **Austin (2008)**	Australia	CBT	Antepartum	Severity of PPD symptoms + PPD diagnosis (State Anxiety, Postpartum anxiety disorder)	-
	**MOTHER-FOCUSED: PSYCHOSOCIAL**					
25.	RCT **Lara (2010)**	Mexico	Psychoeducational strategies + non-directive counseling + supportive interactions via group session	Antepartum	PPD diagnosis + Severity of PPD symptoms (Anxiety symptoms)	-
26.	RCT **Dennis (2009)**	Canada	Supportive interactions via peer support	Postpartum	PPD diagnosis	++
27.	RCT **Ho (2009)**	Taiwan, China	Psychoeducational strategies	Postpartum	PPD diagnosis	-
	**MOTHER-FOCUSED: MIXED**					
28.	RCT **Gao (2015)**	China	IPT + Psychoeducation	Postpartum	Severity of PPD symptoms	++
29.	RCT **Kozinsky (2012)**	Hungary	Group psychotherapy for PPD + IPT + CBT elements + psycho-educational strategies	Antepartum	Severity of PPD symptoms	+
30.	RCT **Gao (2012)**	China	IPT + Routine antenatal education	Antepartum + Postpartum	Severity of PPD symptoms (Perceived Social Support, Maternal role competence)	+

*Note*: Quality assessment based on The Scottish Intercollegiate Guidelines Network (SIGN) criteria for methodological assessment. Key: ++, high quality study designed really well to minimize bias for assessing causality; +, acceptable quality with good enough measures to minimize bias for assessing causality; -, low quality study with poor or no measures taken to minimize bias for establishing causality.

**Table 3:** Study characteristics of mother-child focused interventions.

S. No.	Paper	Location	Intervention	Period of intervention	Key outcomes (Other outcomes)		Quality ^a^
					Maternal	Child	
	**MOTHER-CHILD FOCUSED: PSYCHOLOGICAL**						
31.	Cluster RCT **Rahman (2008)**	Pakistan	CBT	Antepartum + Postpartum	PPD diagnosis	Weight & length (Number of diarrheal episodes + infant immunization)	**++**
	**MOTHER-CHILD FOCUSED: PSYCHOSOCIAL**						
32.	Cluster RCT **Tripathy (2010)**	India	Supportive interactions via participatory group meetings	Antepartum + Postpartum	PPD diagnosis	Neonatal Mortality Rate	**+**
33.	RCT **Cooper (2009)**	South Africa	Psychoeducational strategies + counseling support via home visits	Antepartum + Postpartum	Quality of mother-infant interactions (PPD diagnosis)	Infant attachment	**+**
34.	Cluster RCT **Hennigham (2005)**	Jamaica	Psychoeducational strategies + supportive interactions	Postpartum	PPD scores	Child growth and development	**-**
	**MOTHER-CHILD FOCUSED: MIXED**						
35.	RCT **Stein (2018)** ^b^	UK	CBT + parenting video-feedback therapy (VFT)	Postpartum	PPD diagnosis	Child development + behaviour problems + attachment security	**+**
36.	RCT **Husain (2017)**	Pakistan	CBT + non-directive counseling + supportive interactions	Postpartum	PPD diagnosis	Height and Weight	**+**
37.	RCT **Cooper (2003)**	UK	CBT + Psychodynamic therapy + non-directive supportive counseling	Postpartum	PPD diagnosis + Severity of PPD symptoms (Mental state assessment, quality of mother–infant relationship)	Child development	**+**

^a^ Quality assessment key is described in the note under Table 2.

^b^ Stein’s (2018) study was an RCT comparing two interventions: CBT + VFT vs. CBT + Progressive Muscle Relaxation (PMR). Due to exclusion of exercise-based interventions from this review, we analyzed only the CBT + VFT intervention, thereby treating this trial as a quasi-experimental study reporting on within-group PPD from baseline to end line.

Data extraction was followed by a content analysis of all the interventions. This review presents a descriptive/narrative synthesis of the findings. A meta-analysis of the studies was not possible owing to the heterogeneous nature of the data in terms of PPD outcomes, intervention design, study design, outcome measures and the type of analysis done.

The protocol for the systematic literature review is registered with PROSPERO, International prospective register of systematic reviews (PROSPERO, n.d.). Registration number is CRD42017072706.

## Results

**Fig 1:**
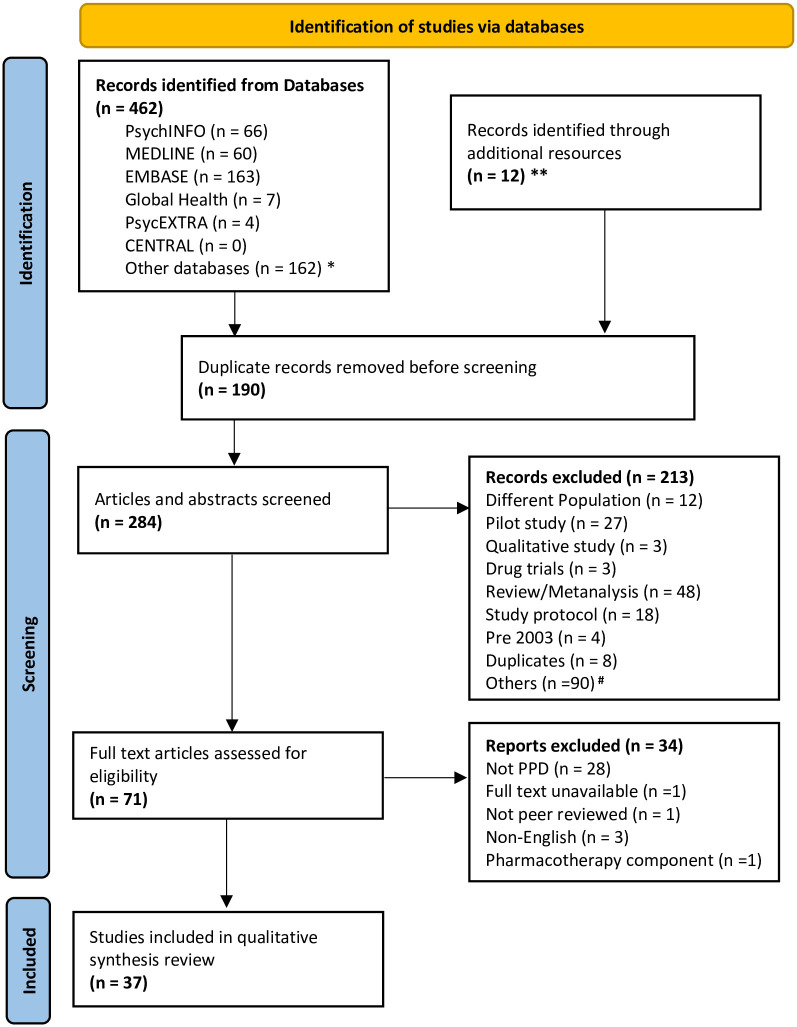
Flow chart showing results of search strategy adapted from PRISMA guidelines. *Includes PubMed and Scopus databases. **Includes records obtained from hand searching of reference lists of the included articles. ^#^ ’Others’ refers to different treatment, not PPD, and/or non-intervention studies.

Fig 1 below presents the results of the database searches (see S1 Table A.1 in S1 File for detailed search strategy).

### Section 1: Study characteristics

The main study characteristics covered here include geographical regions, settings, target population, maternal outcomes and quality of the studies *(See below Table 2: Study characteristics of Mother-focused interventions and Table 3: Study characteristics of mother-child focused interventions)*. About half i.e., 51.3% (n = 19) of these 37 studies were from high-income countries (HICs), 27% (n = 10) came from upper-middle income countries (UMICs) while 21.6% (n = 8) of the evidence was contributed by lower- and middle- income countries (LAMICs). The interventions were delivered across a range of settings, with somewhat equal number of interventions delivered at facilities (n = 16; 43.2%) and within the community (n = 16, 43.2%; including home visits and web-based or telephone/mobile-based accessed at homes). A few of these were delivered at mixed settings, i.e., both in facilities and communities (n = 5; 13.5%). A range of outcome measures were used for assessing PPD and child outcomes across studies and are listed in S1 Tables C.1 and C.2. in S1 File.

The target population varied in terms of whether women were pregnant or had given birth; if they had depression or were at risk during the time of the study. Among the studies targeting pregnant women (n = 18), six enrolled healthy women with no symptoms or history of depression, five studies recruited women at high risk of depression and another seven assessed women with mild to severe depression. Twenty-two studies enrolled mothers who had given birth. Six of these recruited healthy women (child age ranges 0–30 months), 13 trials targeted mothers with symptoms of depression or a major depression diagnosis, and three studies enrolled mothers at high risk of PPD post-delivery.

The maternal outcomes reported by these studies included depressive symptomatology (n = 28) and/or diagnosis of PPD (n = 14). For our review, these outcomes have been clubbed under a single category of PPD. Child growth and child development were the primary child outcomes [13, 19, 36, 42, 46–50]. The screening instruments and other measures related to maternal PPD and child outcomes are listed in S1 Tables C.1 and C.2 in S1 File, respectively.

Generally, the studies were of mixed methodological quality. About 29.7% (n = 11) were given a high quality rating [10, 26, 44, 47, 48, 51–56] while similar number of studies were of acceptable quality (n = 11, 29.7%) [13, 14, 42, 46, 49, 57–62]. The remaining 40.5% (n = 15) studies that were given a low-quality rating [36, 63–76]. 33 trials followed a randomized controlled design with randomization done either at the individual (n = 26) or cluster (n = 7) levels while four applied quasi-experimental design. Allocation concealment was adequately done (i.e., consecutively-numbered, sealed, opaque envelopes containing randomly-generated numbers) in 11 studies [10, 44, 46–49, 51, 53–56] while it was unclear in 13 studies [57, 63–71, 74–76]. The outcome assessors or data analysts were blinded in 13 studies [10, 26, 42, 44, 47–49, 53, 55, 56, 63, 67, 76] while this was unclear in six trials [36, 57, 64, 65, 69, 71] The remaining studies did not use allocation concealment or proper blinding methods. Loss to follow up seemed like a problem with twelve studies of which eight reported greater than 20% loss [13, 19, 46, 57, 69, 73, 74, 77] and four reported attrition rate higher than 40% [62, 63, 71, 76]. ITT analysis was reported and well explained in 20 of the included studies [10, 36, 42, 44, 47–49, 51–56, 62, 63, 66, 72–74].

### Section 2: Key characteristics of intervention approaches

Thirty studies investigated mother-focused interventions (Table 2) while seven studies assessed mother-child focused interventions (Table 3). Both types of interventions were further categorized by the theoretical or therapeutic approach that they applied to improve maternal PPD outcomes (see S1 Tables D.1 and D.2 in S1 File for mother-focused and mother-child focused interventions, respectively).

Generally, the time-points at which the interventions were delivered varied. For instance, most were provided during the postpartum period (n = 17; 44.7%), while some were initiated either in the antepartum phase (n = 9; 23.7%) or were delivered both during pregnancy and after childbirth (n = 11; 30%). A variety of health care providers delivered these interventions across studies. We categorized them into three groups for this review: (a) Health professionals: employed by 20 studies including doctors (n = 3), clinical psychologists (n = 11), nurses (n = 3) and other specialists (n = 6), (b) Paraprofessionals: including midwives (n = 5), government frontline workers (n = 4) or NGO workers (n = 10) delivered the intervention in 20 studies, and (c) Community volunteers were involved in eight studies. Besides these, three studies followed a self-help format using the Internet. The numbers here add up to more than the number of studies reviewed since few employed more than one type of health care providers.

#### Mother-focused interventions

Twenty four of the 30 mother-focused interventions applied psychological strategies [10, 47, 48, 51–55, 58, 60–66, 68–70, 72–76], three used psychosocial ones [44, 67, 71] and three were mixed interventions [56, 57, 59].

*Psychological approaches*. Amongst the psychological interventions, seventeen were developed based on CBT principles [10, 51, 54, 58, 60, 62, 63, 65, 66, 68–70, 72–76]. Integral to the CBT approach, the cognitive restructuring component was practiced by all to identify and replace unhelpful thoughts with more helpful ones. Adding to this was the behavioural component that comprised scheduling pleasant events, relaxation, goal setting, problem solving, assertion skills, and building broad social support network [10, 26, 42, 63, 69]. Furthermore, CBT was adapted based on mode and method of delivery. For instance, six studies evaluated group-based CBT [54, 63, 69, 72, 73, 76] while in nine, CBT was delivered individually [10, 51, 58, 60, 62, 66, 70, 74, 75]. The different methods of delivery included web- or app-based platforms by four recent studies [58, 60, 62, 66], telephonic delivery [70], audio-visual methods [64, 65, 69] and through home visits [10, 76]. Furthermore, three studies explored the impact of the type of health worker delivering the CBT on maternal PPD outcomes [48, 51, 74].

Besides CBT, four trials examined Behavioural Activation (BA) techniques [52, 55, 61, 77] BA emphasizes identifying and reinstating previously liked behaviours that had now decreased or were being avoided [17, 78, 79]. One of these evaluated an internet-based BA [61]. The two more recent trials [53, 55] investigated an intervention called the Thinking Healthy Program Peer-delivered (THPP) primarily adapted from CBT based Thinking Healthy Program (THP) [18, 80]. Their adaptation focused more on its BA component to make it deliverable by non-specialists and hence, will be considered essentially as BA interventions in this systematic review. Yet another recent trial [48] examined BA, but as part of a multicomponent task-sharing psychological intervention that incorporated several elements including problem solving, psychoeducation, health thinking adapted from the THP [18], relaxation and birth preparation. Of note, CBT and BA are mostly similar except for a major divergence from cognitive restructuring that is not originally allowed in BA [81]. One fairly recent study compared what they called a high intensity treatment (HIT) having Problem Solving Treatment (PST) as its core component with Enhanced Usual Care (EUC) comprising of a low-intensity treatment (LIT) [World Health Organization Mental Health Gap Action Programme (mhGAP)] [82] for perinatal depression. The PST element entailed guiding the mother to divide her present psychosocial difficulties into smaller parts, find solutions for them and attempt them to resolve these problems while utilizing one’s own as well as available social support [47].

*Psychosocial approaches*. The key strategies evaluated by psychosocial mother-focused interventions [44, 67, 71] were psychoeducation, non-directive counseling and supportive interactions. Two out of three were delivered face to face in a group format while one was delivered individually via telephonic sessions [44]. The latter was also the only one amongst psychosocial approaches that investigated a peer support intervention.

*Mixed approaches*. Mixed interventions combined both psychological and psychosocial element to address PPD. In our review, all three mother-focused mixed interventions comprised Interpersonal Therapy (IPT) as the psychological component clubbed with the psychosocial component of supportive interactions [56, 57, 59]. IPT for PPD links presence of depressive symptoms to distress arising from a mother’s relationships with her child, partner, family, etc. and is mostly caused by negative or maladaptive communication styles as per the interpersonal theory [12, 83]. Thus, IPT addresses these maladaptive patterns assuming that improved interpersonal relations will help bring positive changes to PPD [12].

#### Mother-child focused interventions

With mother–child dyad as their main target, these interventions [13, 26, 36, 42, 46, 49, 50] focused on increasing maternal knowledge around child’s growth and development, improving child rearing practices, mother-infant relationship and maternal health.

*Psychological approaches*. Fitting under this category was only one study [26] wherein the intervention was called THP [18]. It applied ‘low intensity’ cognitive restructuring and behavioural activation and employed rural non-specialist community health workers to deliver it.

*Psychosocial approaches*. Three studies evaluated psychosocial interventions [36, 46, 50] comprising similar components as those of mother-focused interventions described above. In addition, a rather commonly practiced strategy that stood out was psychosocial stimulation [36, 46]. It aimed at sensitizing the mother to her infant’s needs and capacities so as to have sensitive, responsive interactions with her infant. All three of the psychosocial studies were community based, delivered either at participants’ homes or in community group settings.

*Mixed approaches*. The three mixed interventions [13, 42, 49] primarily included CBT as the psychological component plus non-directive counseling as the psychosocial one. One of these [14] also examined psychodynamic therapy as the psychological component. All three studies applied cognitive behavioural elements primarily to address mother’s issues related to infant management and interaction rather than maternal depression. Psychodynamic components worked towards understanding the mother’s portrayal of her infant and their relationship by exploring her attachment history [13, 84]. The psychosocial components targeted strengthening support and improving quality of mother-child interactions. The third study used video feedback and incorporated parenting components that entailed working on increasing mother’s responsiveness by attending to her child’s signals, provision of emotional support and sensitivity towards her child’s attachment needs [49].

Summation of the above evidence indicates a preference for psychological approaches within the mother-focused interventions. In contrast, mother-child focused interventions seemed more inclined towards psychosocial and/or mixed approaches. Furthermore, CBT emerged as the most frequently practiced and an effective psychological technique for PPD. Techniques such as BA too came forth as an equally good option though the studies evaluating these were small in number. As for the psychosocial and mixed strategies, all the components were equally practiced and the evidence did not demonstrate any one preferred technique.

### Section 3: Maternal and child outcomes

#### Mother–focused interventions: Maternal outcomes

*Mother-focused*: *Psychological approaches*. Of the 24 studies using a psychological approach, a CBT based intervention package including both cognitive restructuring and behavioural components was associated with a significant impact on maternal PPD outcomes compared to the treatment as usual (TAU) in 15 studies [10, 51, 54, 58, 60, 62, 64–66, 68–70, 73, 74, 76]. However, the quality of these studies varied (Table 2). All four web-based CBT interventions [58, 60, 62, 66] reported statistically significant differences in PPD diagnosis. However, one of these could not ascertain the effectiveness of the intervention due to lack of an active control group [62]. Furthermore, three of these especially highlighted the advantages of having a therapist or a supportive person maintaining regular contact with the participant on improving maternal PPD outcomes [60, 62, 66]. Notably, all of these were evaluated with a small sample size and their greatest pitfall was participants’ access to alternative treatments, which could possibly have yielded exaggerated effect sizes. Next, there were five group-based CBT interventions [54, 63, 69, 73, 76] that demonstrated mixed results with only half of the studies [69, 76] showing a significant decline in severity of PPD over a longer period of time. One of these achieved such an impact only in low-moderate risk women and for African-American women at high-risk [69]. Specifically, a home visiting CBT package was associated with significantly reduced likelihood of receiving a PPD diagnosis in two studies [10, 76]. Three quasi-experimental studies that evaluated CBT [64, 65, 68] also produced significant results although their interpretation requires caution owing to the lack of a robust study design.

Besides CBT, an intervention package including BA techniques [52, 53, 55, 61] was also associated with significant impact on maternal PPD. Of these, the shorter version of BA [52] generated clinically significant improvement in PPD at 3-months postpartum (p = 0.04). The Internet based BA, called the ‘Netmums’, though recorded higher attrition rates [61, 77]. The two more recent ones investigating a peer-delivered BA intervention [53, 55] generated somewhat mixed results. Both demonstrated significant reduction in PPD symptom severity at three months and overall improvement in recovery (PHQ9 score <5 at both 3 and 6 months). However, the results for primary outcomes at six months were unfavorable and varied between the two arms. While both sites were associated with non-significant reduction in PPD symptom severity, only the Indian arm reported significantly higher prevalence of remission (p = 0·04), at six months. One study that examined BA as part of a task-sharing psychological intervention [48] found it to be ineffective in producing a positive impact on response to treatment (3 and 12 months postpartum) or recovery (at 12 months postpartum) though they did observe considerably reduced mean EPDS scores in the intervention arm at both time-points. Similarly, the PST intervention [47] was unable to demonstrate its effectiveness over EUC in terms of recovery from PPD at 6 months post-delivery. Though, it did produce significant positive results for those with severe PPD. To add here, the last two studies mentioned here were also the only mother-focused ones that also investigated child outcomes.

*Mother–focused*: *Psychosocial approaches*. The results of the three mother-focused psychosocial approaches [44, 67, 71] were mixed though they constituted similar components. Only one study [44], the peer support intervention reported high quality evidence for significantly lesser likelihood of mothers to develop PPD symptoms at 12 months postpartum (P = 0.02), though the study reported nonsignificant results at 24 weeks.

*Mother–focused*: *Mixed approaches*. A mixed intervention package combining IPT and psychoeducational strategies was linked with significant changes in PPD outcomes in three studies [56, 57, 59] with one of these having additional elements of group psychotherapy and CBT [59]. Two of these examined the same intervention package though one was delivered in groups [57] and the other individually [56]. However, the attrition rate was quite high (26.8%) in the group-based one.

#### Mother–focused interventions: Child outcomes

*Mother-focused*: *Psychological approaches*. Only two of the 30 mother-focused interventions that applied a psychological approach examined child outcomes. Both these interventions, the PST one [47] and the task-sharing multicomponent one [48] were unable to demonstrate a significant impact on any of the child outcomes. In the former study, infants in the intervention arm did not differ substantially in any of the anthropometric measures or cognitive and motor development. The latter one too showed similar results for child growth as well as for other birth and child health outcomes including complete immunization, diarrhoea and respiratory infections (Table 4). (see S1 Table E.1 in S1 File for detailed statistical findings)

**Table 4:** Maternal and child outcomes for mother-focused interventions.

S. No.	Study	Intervention; Period; Duration	Maternal PPD Outcomes* [Child Outcomes]
	**Mother-Focused: Psychological**		
1.	Quasi Experimental **Huynh-Nhu Le (2021)**	CBT; Antepartum + Postpartum; Six weeks	**(a) Non-significant reduction in PPD scores** across groups at any time point; **(b) Significant reduction in PPD scores** for (a) completers, from T1 to T2 and from T1 to T3 with a non-significant decrease from T2 to T3; **(c) Non-significant reduction in PPD** scores for the non-completers; **(d) Significant reduction in PPD scores** for zero-class participants from T1 to T3, and from T2 to T3, with no significant change from T1 to T2. **(e) Significant reduction in** PPD Scores from T1 to T3 for all participants
2.	RCT **Jannati (2020)**	CBT; Postpartum; 8 weekly	**(a) Significant reduction in PPD scores** for both intervention and control groups **(b) Significant difference in PPD scores** between intervention and control groups
3.	Cluster RCT **Ngai (2019)**	CBT; Antepartum + Postpartum; 3 sessions each at 2 and 4 weeks postpartum	**(a) Significant difference in PPD scores** (measured in terms of low risk of PPD on EPDS) indicating group-by-time interaction effects on risk of PND at 6 weeks postpartum **(b) Non-significant difference in PPD scores** at 6 and 12 months
4.	Cluster RCT **Gureje (2019)**	PST; Antepartum + Postpartum; 8 weekly sessions each at antepartum and postpartum	**(a) Non-significant reduction in PPD scores** (remission of PPD) at 6 months postpartum **(b) Significant reduction** in PPD scores for severe PPD **[Child Outcomes**: **(a) Non–significant improvements** in child growth and development; **(b) Significant increase** in exclusive breastfeeding]
5.	RCT **Lund (2019)**	Task-sharing multicomponent; Antepartum + Postpartum; Six weekly sessions (45–60 min)	**(a) Non-significant reduction** in PPD scores at 3 and 12 months postpartum **(b) Non-significant improvement** in recovery at 12 months postpartum **[Child Outcomes**: **(a) Non–significant improvements** in child growth and development; **(b) Non-significant reduction** in diarrheal episodes or respiratory infections **(c) Non-significant increase** in the likelihood of complete immunization]
6.	RCT **Fuhr (2019)**	BA; Antepartum + Postpartum; 6–14 sessions over 7–12 months	**(a) Non-significant reduction** in PPD scores at 6 months **(b) Significant decrease** in PPD prevalence (or higher prevalence of remission) at 6 months **I Significant reduction** in PPD scores at 3 months **(d) Non-significant decrease** in PPD prevalence (or prevalence of remission) at 3 months **(e) Significant decrease** in overall prevalence of PPD (improved recovery, i.e., PHQ9 score<5 at 3 and 6 months postpartum)
7.	RCT **Sikander (2019)**	BA; Antepartum + Postpartum; 10 sessions– 4 antepartum, 6 postpartum	**(a) Non-significant reduction** in PPD scores at 6 months **(b) Non-significant decrease** in PPD prevalence (or prevalence of remission) at 6 months **(c) Significant reduction** in PPD scores at 3 months **(d) Significant decrease** in PPD prevalence (or prevalence of remission) at 3 months **(e) Significant decrease** in overall prevalence of PPD (improved recovery, i.e., PHQ9 score<5 at 3 and 6 months postpartum)
8.	RCT **Dimidjian (2017)**	BA; Antepartum; 10 sessions, flexible	(a) **Significant reduction** in PPD scores averaged across follow-up time points (b) **Clinically significant improvement** for BA at 3-months postpartum
9.	RCT **Forsell (2017)**	CBT; Antepartum; 10 weekly sessions	(a) **Significant reduction** in in PPD scores post-treatment (b) **Significant improvement** in PPD symptoms post-treatment (c) **Significant decrease** in PPD prevalence post treatment
10.	RCT **Pugh (2016)**	CBT; Postpartum; Seven weekly modules	(a) **Significant reduction** in PPD scores at 10 weeks follow-up and at 4 weeks follow-up post treatment completion
11.	RCT **Milgrom (2016)**	CBT; Postpartum; 6 weekly sessions	(a) **Significant reduction** in severity of PPD symptoms at 12 weeks follow-up (b) **Significant reduction** in PPD prevalence at 12 weeks follow-up
12.	RCT **Jesse (2015)**	CBT; Antepartum; 6 weekly sessions, 2 hours each	(a) **Non-significant reduction in** PPD scores (b) **Significant reduction** in PPD scores in women at low-moderate risk (c) **Significant reduction** in PPD scores for at-high-risk African-American
13.	Quasi-experimental **Ashtiani (2015)**	CBT; Antepartum; 8 sessions, 40–60 min	(a) **Significant reduction in** average PPD scores at 2 weeks postpartum
14.	Quasi experimental **Dimidjian (2015)**	MBCT; Antepartum; 10 series of eight 2 hour sessions each	**(a) Significant reduction** in PPD scores with sustained decrease during the intervention
15.	RCT **Tandon (2014)**	CBT; Antepartum + Postpartum; 6 weekly sessions	(a) **Significant reduction in** PPD scores at 1 week, 3 months and 6 months post intervention **(b) Non-**s**ignificantly lower** PPD incidence at 6 months post-intervention
16.	RCT **Jiang (2014)**	CBT; Postpartum; 40 mins session/week (no. of weeks unclear)	(a) **Significant reduction** in PPD scores at 6 months postpartum
17.	Quasi-experimental study **Hou (2014)**	CBT + SFT; Postpartum; 3 months (CBT– 13 weekly sessions; SFT– 6 fortnightly sessions)	(a) **Significant reduction** in PPD scores post intervention and at 6-, 12-, 18- and 24-months follow-up after intervention
18.	RCT **Pinheiro (2013)**	CBT and RCT; Postpartum; Not mentioned	(a) **Non-significant reduction** in PPD scores at 12 months after intervention
19.	RCT **Ammerman (2013)**	CBT; Postpartum; 15 weekly + 1 booster sessions	(a) **Non-significant** reduction in PPD scores (b) **Significant reduction** in PPD prevalence post-treatment and at 3 months follow-up post-treatment
20.	RCT **O’Mahen (2013)**	BA; Postpartum; 11 weekly sessions	(a) **Significant reduction** in in PPD scores for non-depressed participants (b) **Significant reduction** in PPD scores in individuals with mild–moderate depressive symptoms (c) **Significant reduction** in PPD scores in individuals with more severe depressive symptoms
21.	RCT **Milgrom (2011)**	CBT; Postpartum; 6 weekly sessions + 3 GP visits	(a) **Non-significant reduction** in PPD scores across different treatment groups (b) **Significant increase** in PPD prevalence in GP management group
22.	RCT **Le (2011)**	CBT; Antepartum + Postpartum; 8 weekly + 3 booster sessions	(a) **Significant reduction in** PPD scores at time point 2 (late pregnancy) (b) **Significantly lower incidence** of moderate depression at time point 2 (late pregnancy)
23.	Cluster RCT **Brugha (2010)**	CBT or person centered; Postpartum; 8 weekly sessions	(a) **Significant reduction** in PPD scores at 6 months postpartum
24.	RCT **Austin (2008)**	CBT; Antepartum; 6 weekly + 1 follow-up sessions	(a) **Non-significant reduction** in PPD scores at 6 weeks post-intervention, 2 months postpartum and 4 months postpartum (b) **Non-significant reduction** in PPD prevalence
	**Mother-Focused: Psychosocial**		
25.	RCT **Lara et al (2010)**	Psychoeducational, non-directive counseling, supportive interactions via group sessions; Antepartum; 8 weekly sessions	(a) **Non-significant reduction** in PPD scores (b) **Significantly lower** cumulative incidence of major PPD
26.	RCT **Dennis (2009)**	Supportive interactions via peer support; Postpartum; 4 sessions + needs based sessions	(a) **Significant reduction** in PPD scores at 12 weeks postpartum (b) **Non-significant reduction** in PPD scores at 24 weeks postpartum
27.	RCT **Ho (2009)**	Psychoeducational strategies; Postpartum; Unclear	(a) **Non-significant reduction** in PPD scores at 6 weeks and at 3 months postpartum
	**Mother-Focused: Mixed**		
28.	RCT **Gao (2014)**	IPT + Psychoeducation; Postpartum; 2 sessions	(a) **Significant reduction** in PPD scores at 6 weeks postpartum
29.	RCT **Kozinsky (2012)**	Group therapy + IPT + CBT elements + psycho education; Antepartum; Four weekly sessions	(a) **Significant reduction** in PPD prevalence 6 weeks postpartum
30.	RCT **Gao (2011)**	IPT + Routine antenatal education; Antepartum + Postpartum; 2 weekly + 1 follow-up sessions	(a) **Significant reduction** in PPD scores at 3 months postpartum

^a^ PPD outcomes include: (1) PPD diagnosis made by clinicians using diagnostic tools (e.g., SCID-IV, MINI), (2) Severity of PPD symptoms, i.e., having some features of depression but not the full list to qualify for a diagnosis.

To sum up, the maximum improvements in maternal PPD seemed to be brought about by mixed approaches than by psychological and psychosocial ones alone. The commonest psychological component amongst the mixed approaches was IPT while supportive interactions were the common psychosocial ingredients. Psychological interventions were more practiced than the other two and were also quite effective. Amongst these, besides the CBT techniques that were associated with significant reductions in PPD symptoms, BA techniques also seemed equally effective though fewer studies investigating the impact of BA were included in this review. The psychosocial approaches, on the other hand, were associated with poor PPD outcomes.

#### Mother-child focused interventions: Maternal outcomes

*Mother-child focused*: *Psychological approaches*. Even amongst the mother-child focused studies, the only psychological intervention package of CBT [26] was associated with significantly lesser likelihood to have maternal PPD at 6 months (P< 0.0001) as well as at 12 months postpartum (P < 0.0001). Other advantages of this package included experiencing lower levels of disability, improved overall functioning and increased perceived social support at follow-up assessments than the control group.

*Mother-child focused*: *Psychosocial approaches*. Two studies [36, 46] evaluating home-visiting packages were linked with significant changes in maternal PPD. Of these, the intervention package comprising of psychoeducation and supportive interactions [36] brought significant decline in maternal PPD. Number of home-visits also seemed to play a substantial role with 40–50 home visits showing the greatest impact while <25 home visits appeared to have no effect.

The psychoeducation cum counseling package delivered in a South African region [46] too was related with a significant change in the severity of PPD symptoms (i.e., lower EPDS scores) but at six months only (P = 0.04). However, it did not demonstrate any impact on PPD prevalence. On the other hand, a third study evaluating a community-based package composed of supportive interaction via participatory group meetings [50] was not linked with any significant impact on PPD until the third year of the trial when they demonstrated marked reductions in moderate PPD only.

*Mother-child focused*: *Mixed approaches*. Inclusion of CBT as a psychological component to a mixed intervention package seemed to be associated with significant reduction in maternal PPD in three studies [14, 42, 49]. One of these called the LTP Plus [42] showed significant reductions in maternal PPD at 3 months postpartum and sustained at 6 months postpartum. In the other study [13] evaluating the benefits of three different treatment conditions, only psychodynamic therapy was associated with considerable decline in PPD (SCID-IIIR) [13, 14]. Interestingly, none of these interventions found any apparent long-term (9 months postpartum) benefits for PPD. The only exception was the third one that delivered VFT+CBT [49] and reported marked within-group decrease in PPD levels from baseline. About 80% of participants no longer received a PPD diagnosis and sustained this improvement at 2 years postpartum (85% remission rates) (Table 5). For detailed statistical findings please see S1 Table E.2 in S1 File.

**Table 5:** Maternal and child outcomes for mother-child focused interventions.

S. No.	Study	Intervention; Period; Duration	Maternal PPD Outcomes ^a^	Child Outcomes
	**Mother–child focused: Psychological**			
31.	Cluster RCT **Rahman (2008)**	CBT; Antepartum + Postpartum; 16 sessions– 4 antepartum and 12 postpartum	(a) **Significant reduction** in PPD prevalence at 6 and 12 months postpartum	(a) **Non-significant** reduction in infant stunting or malnutrition (b) **Significant reduction** in diarrheal episodes at 12 months of child’s age (c) **Significant increase in** the likelihood of complete immunization of infants at 12 months of child’s age
	**Mother–child focused: Psychosocial**			
32.	Cluster RCT **Tripathy (2010)**	Supportive interactions via participatory group meetings; Antepartum + Postpartum 20 monthly sessions	(a) **Non-significant reduction** in PPD scores (b) **Significantly lower** incidence of moderate depression in year 3 of the study	(a) **Significant reduction** in NMR
33.	RCT **Cooper (2009)**	Psychoeducational strategies + counseling support via home visits; Antepartum + Postpartum; 16 visits till 5 months of child’s age	(a) **Significant reduction in** PPD scores at 6 months postpartum only (b) **Significant increase** in mother’s sensitivity at 6 and 12 months postpartum **(c) Significant reduction** in intrusiveness of mothers at 6 and 12 months	(a) **Significant increase in** more securely attached infants at 18 months of child’s age
34.	Cluster RCT **Henningham (2005)**	Psychoeducational strategies + supportive interactions; Postpartum; Weekly one hour visit over one year	(b) **Significant reduction** in PPD scores **Largest reduction** for 40–50 home visits **Lesser reduction** for 25–39 home visits N**on-significant reduction** for 0–24 home visits	(a) **Non-significant difference** for child development (b) **Significant correlation** between final PPD and DQ scores in boys only (c) Child Growth: Not reported
**Mother–child focused: Mixed**				
35.	RCT **Stein (2018)** ^b^	CBT + VFT; Postpartum; 11 visits—6 weekly and 5 fortnightly	**(a) Non-significant reduction** in PPD scores at 1 and 2 years’ time points	**(a) Non-significant difference** for child development and behavioural outcomes
36.	RCT **Husain (2017)**	CBT + non-directive counseling + supportive interactions; Postpartum; 10 weekly sessions	(a) **Significant reduction** in PPD scores at 3 months (sustained at 6 months)	(a) **Non-significant difference** in height and weight measure
37.	RCT **Cooper & Murray (2003)**	CBT + Psychodynamic therapy + non-directive supportive counseling; Postpartum; 10 weekly sessions	(a) **Non-significant reduction** in PPD scores for all three treatments at 4.5 months postpartum only (b) **Significant reduction** in PPD scores in psychodynamic therapy group only (c) **Non-significant reduction** in PPD prevalence at 5 years postpartum	(a) **Significant reduction** in emotional and behavioural problems in infants for non-directive counseling at 18 months postpartum only (b) **Non-significant improvement** in management of infant behaviour, mother-infant attachment and infant cognitive development at 5 years

^a^ The PPD outcomes include: (1) PPD diagnosis made by clinicians using diagnostic tools such as SCID-IV or MINI (2) Severity of PPD symptoms, i.e., having some of the features of depression but not the full list to qualify for a diagnosis.

^b^ Treated as quasi-experimental study in this review. Explained in details above in the notes under Table 3.

#### Mother-child focused: Child outcomes

*Mother-child focused*: *Psychological approaches*. The THP intervention package comprising CBT [26] reported a non-significant impact on primary outcomes of infant stunting and malnutrition, though it showed significant effects on other secondary child outcomes, such as, reduction of diarrheal episodes and increased likelihood of full immunization at 12 months.

*Mother-child focused*: *Psychosocial approaches*. The counseling support via home visits with women in the third trimester [46] was associated with significantly increased sensitivity amongst infants both at 6 months and 12 months post treatment. In addition, the intervention also resulted in marked increase in secured attachments at 18 months. One of these studies that looked into the impact of a community participatory intervention on Neonatal Mortality Rate (NMR) [50] was associated with substantially bringing down the overall NMR by 32% in intervention clusters and by 45% in years 2 and 3. The home-based psychosocial intervention constituting psychoeducational strategies and supportive interactions [36] was non-significantly correlated with developmental quotient for both boys and girls.

*Mother-child focused*: *Mixed approaches*. The three mixed interventions were associated with non-significant impact on child growth [42], emotional, behavioural or developmental outcomes [14, 49]. Amongst these, the major trial that compared three treatment conditions reported significantly more sensitive early mother-child interactions only for the non-directive counseling component at 18 months postpartum [14]. No significant differences were found for child’s cognitive development at the age of five years (P = 0.91). Similar findings came from the CBT+VFT intervention with no evidence of the treatment impact on primary outcomes including child’s cognitive and language development, behavioural problems and secure attachments [49].

In summary, results from the seven mother-child-focused interventions present a mixed picture. Whereas, the psychological approach did not have significant impact on primary outcomes of child growth, it was able to bring positive changes to other child health outcomes. Moreover, it reduced rates of maternal PPD significantly. Two out of three psychosocial interventions demonstrated significant impact on very different child outcomes. One was able to reduce NMR and the other showed improvements in secure attachments. The former study however, did not change maternal PPD outcomes immediately but only in the last stages of the study, while the latter reduced the severity of PPD symptoms substantially at the first time-point. A relevant and a fascinating observation here is that the performance of the psychosocial interventions, apparently, become better for PPD outcomes once child related components were added to the intervention package. Somewhat likewise, the three mixed interventions, although did not report improvements in child outcomes, yet all were able to demonstrate positive changes to maternal PPD.

Overall, in terms of service providers, amongst the 15 mother-focused psychological interventions associated with significant improvements in PPD outcomes, seven (47%) were delivered by specialists [10, 51, 60, 62, 64, 66, 76], three (20%) by non-specialists [53, 70, 73], and another five (33%) by a combination of the two [52, 61, 65, 68, 69]. The one psychosocial intervention that significantly reduced the likelihood of developing PPD symptoms was provided by lay peer counsellors [44]. Two of the three mixed interventions producing significant positive results had non-specialists such as midwives as their delivery agents [56, 57] while one employed a combination of both including doctors, psychiatrists and lay health visitors [59]. The mother-child focused interventions seemed to be mostly delivered by lay community health workers [26, 36, 46]. Rather, and as somewhat expected, specialists were not the service providers for any of the psychosocial interventions. On the other hand, the three mixed interventions presented varied results. Of the two studies that employed specialists to deliver their interventions, while one was unable to prove its effectiveness on both maternal and child outcomes [49], the second one significantly improved only the PPD outcomes [13]. To point out here, the positive PPD results were evident only for the psychodynamic treatment group. Similar findings as the latter trial were reported by the third mixed intervention that utilized a heterogeneous group of service providers [42].

Notably, amongst mother-focused interventions, majority of those applying psychological techniques were evaluated in HICs [14/24, (58.3%)], of which eleven (78.6%) brought significant improvements in PPD. Five of these came from the UMICs [5/21, (24%)], of which three (60%) were associated with a significant impact on PPD. The one psychosocial mother-focused intervention indicating effectiveness was from HIC as well while the three mixed ones showing a marked improvement in PPD hailed from UMICs. Strikingly, only three out of 30 mother-focused interventions included in this review came from LAMICs. It might be of some interest that all three of these interventions utilized a psychological approach. Conversely, only two out of the seven mother-child-focused interventions were contributed by HICs while the rest of the studies were equally spread across other income regions.

## Discussion

This research paper systematically reviews 30 mother-focused interventions and 7 mother-child focused interventions. We would like to draw the reader’s attention towards the huge disparity between the number of studies under the two kinds of intervention with very few studies on mother- child focused interventions included in this review. This difference is mainly due to availability of larger body of literature on mother-focussed intervention and a limited number of studies that examine interventions integrating both maternal and child components, especially with a focus on PPD. This also highlights a gap in literature and is one of the aspects that needs to be balanced in future research.

Overall, in our review, significant reduction in maternal PPD outcomes was brought by 21/30 (70%) mother-focused interventions and by 4/7 (57%) mother-child focused interventions. These findings strongly favor the mother-focused interventions in improving maternal PPD outcomes. One plausible explanation for this difference could be that the mother- focused interventions primarily emphasized on improving woman’s PPD alone by managing negative thoughts, moods and behaviours using psychological approaches such as CBT and BA. On the other hand, most mother-child focused interventions were more concerned with improving dyadic interactions and child health outcomes while expecting subsequent improvements in mother’s PPD.

Our analysis further suggests that amongst the mother-focused interventions, the mixed approaches performed considerably well in reducing PPD symptoms [56, 57, 59] than psychological and psychosocial ones alone. A noteworthy observation here is that IPT was the common psychological ingredient across all mixed approaches while psychoeducation was the common psychosocial component. In support, the existing evidence has indicated IPT to have immense promise as a treatment for PPD with a few reviews even suggesting it’s efficacy to be higher than other psychotherapies [9, 12, 83, 85]. Since, in our review, IPT was delivered combined with psychoeducation and CBT, it might be less than reasonable to completely attribute the effects on PPD outcomes to this technique.

Amongst the psychological mother-focused techniques that followed closely in significantly improving maternal PPD, the CBT components of cognitive restructuring and behavioural modification were the active ingredients that seemed to bring about substantial improvements. These findings are consistent with previous trials and meta-analyses [1, 2, 5, 86, 87] wherein, CBT was shown to be effective in treating general depression as well as PPD. Backing our finding further is another trial from rural Pakistan where the LTP program delivered without the CBT components, though, brought about significant changes in infant rearing practices and knowledge of mothers, was however, unable to change maternal mood [19]. However, one need not ignore the fact that since CBT has the widest evidence base and is frequently investigated more than the other approaches, it might not be always correct to attribute maximum positive changes in PPD to CBT alone [88]. Besides CBT, the other psychological approaches like the BA demonstrated similar positive effects on PPD. The literature too has often touted BA as ‘non-inferior’ to both CBT and pharmacotherapy for depression [78, 79]. An advantage of focusing on BA is that it seems easily deliverable by non-specialists who sometimes might find the cognitive restructuring component of CBT as complex and difficult to deliver within the community [53, 55, 80]. This could have important implications especially from a pragmatic intervention development and implementation point of view.

This not so apparent difference between the various psychological techniques emerging from our findings concurs with the existing evidence that all hold promise as effective treatment options for PPD [1, 2, 78]. However, since the studies evaluating other approaches besides CBT such as BA, IPT and psychodynamic therapies were few in number and the latter two were evaluated as part of mixed strategies, we suggest caution while interpreting these findings and to explore further evidence that compares each one’s effectiveness empirically. Furthermore, this might also imply that factors extraneous to the therapeutic technique might be exerting some influence on uptake and overall impact of the intervention [78, 89]. One such factor often emphasized by various studies reviewed here [10, 13, 26, 36] and elsewhere [2, 3, 37], is prolonged intervention delivery plus long-term follow-ups. In our review, a community-based intervention [36] found more number of home visits as a predictor for degree of improvement in PPD outcomes. This is in line with CBT’s basic premise, specifically, to give sufficient time to treatment so as to learn managing one’s emotions and thoughts well enough [41, 88]. Strengthening our discussion further, authors of another study [48] attributed the non-success of their task-sharing multi-component psychological intervention to several likely reasons including the small number of sessions (6 vs. 16 sessions in the original THP [90]). On top of this, they further added that these few sessions comprised of a variety of components including PST, BA, psychoeducation and cognitive reframing, thereby leaving the mothers with very little time and space to comprehend and apply many new skills. Although, a few earlier meta-analyses reported no significant association between length of CBT and its impact on depression, yet, they found that 14.9 weeks or more of CBT on an average was associated with a decline in general depressive symptoms [2, 87]. Similarly, in the IPT literature, 16–20 sessions are the usual length of therapy and many strongly recommend that delivering IPT sessions over a span of a year might increase its effectiveness manifold [12, 41]. Others, too, have considered length of the intervention as a plausible contributing factor and have suggested conducting further investigation into its possible associations with significant long-term impacts on PPD as well as child outcomes [26, 37, 50].

Now, amongst the psychosocial approaches, interestingly, the mother-focused ones performed averagely with only third of these [44] producing a significant though short-lived impact on PPD outcomes. One may jump to attributing the non-performance of the other two [67, 71] to the use of group-based modality based on the existing literature that suggests that attending groups might not be easy for both mothers and pregnant woman [91], which could result in higher dropout rates [71, 85]. However, it might not be entirely true in our case. Other factors such as small sample size [71] and a weak study design [67] could also have contributed to these negative findings. To add to this, the literature regarding effectiveness of group therapy for PPD has been rather inconclusive [2, 5, 85]. Nevertheless, these findings do make it difficult to confidently pinpoint at some definitive psychosocial ingredients that can be generally responsible for improving PPD. On the other hand, the review of mother-child focused psychosocial interventions indicates that adding child related elements improved the performance of psychosocial approaches in alleviating PPD to quite an extent. This finding is fascinating and quite relevant especially in terms of informing design of integrated maternal and child interventions. It is important to emphasize here that all three of these interventions had mother’s depression as their primary focus. Furthermore, supportive interaction was the commonest strategy amongst these interventions that seemed to be linked with improvement in maternal PPD outcomes [13, 36, 50]. These align with the inference that additional social support should benefit postpartum women [40, 44], which is based on earlier meta-analytic results that highlight lack of social support as one of the many social determinants associated with increased risk of PPD [8, 92]. Moreover, in terms of child outcomes, these approaches seemed to perform best amongst the mother-child focused interventions.

As mentioned earlier, the mixed mother-focused interventions improved PPD [56, 57, 59], whereas, the mixed mother-child focused ones [13, 14, 49] generated varied results. Of the latter, the one that showed positive effects on mother-child interactions and child behavioural problems was unable to have an impact on PPD outcomes [13]. It could be ascribed to the fact that in this study, CBT was not primarily directed at PPD but at problems identified by mother in infant management and quality of mother–infant interaction. In a later study [46], the same authors set out to investigate the impact of their intervention on mother-child relationship and whether it will also bring about a positive change in maternal depression. Their intervention, though, demonstrated improved mother-infant interaction, secure attachment and higher maternal sensitivity, it did not improve maternal mood. Likewise, an earlier systematic review of the treatments for mothers and infants [7] concluded that interventions targeting mother-infant relationship can be potentially effective for ameliorating child outcomes and increase mother’s sensitivity towards their child while having no impact on her PPD [7, 93]. Similarly, in Bangladesh, a psychosocial stimulation plus food supplementation intervention reported beneficial growth and development effects for malnourished children with no reductions in mother’s PPD six months later [94]. Contrarily, the LTP Plus intervention while reduced depressive symptoms significantly; the child growth outcomes remained unaffected [42]. Similarly, the other mixed mother-child focused intervention exploring the benefits of a CBT+VFT on enhancing mother-infant interactions also had a substantial impact on PPD outcomes but none on child developmental ones [49]. A point worth noting here would be the non-exclusion of participants on antidepressants that might have affected PPD symptom severity. However, since we are only considering one specific intervention from this trial and comparing pre- and post-test PPD scores, we advise exercising caution while interpreting causality.

To add further, any alterations in mother’s PPD did not apparently predict variations in child’s growth and developmental outcomes [26, 36, 50]. A recent review of the literature [39] and an earlier systematic review [34] that analyzed eight RCTs aimed at treating PPD through targeting the mother–infant relationship, both concur with our findings. They suggested that improvements in maternal mood might be necessary but not sufficient to improve additional dyadic and/or child developmental outcomes alone.

Another feature of our review was the inclusion of studies from both HICs and LMICs, which generated a few interesting findings. A noticeable difference in the evidence from the different income groups was the increased use of Internet based therapies in studies mainly from HICs. Four such mother-focused interventions [60, 62, 66, 77] using web-based psychological techniques of CBT and BA produced a significant impact on maternal PPD outcomes. One could attribute this difference to comparatively higher advancements in technology in HICs and access to Internet within homes amongst majority of their population [16, 95, 96]. On the contrary, a similarity amongst the different income regions reflected through our findings was the shift towards home-based intervention delivery by non-specialist workers, which seemed to decrease PPD symptoms in both HICs and LAMICs including the UK [13], the USA [10], Jamaica [36], India [53], Pakistan [19, 55], and South Africa [46]. This shift could be attributed to the kinds of barriers to accessing treatment faced by HIC’s and LAMICs alike and may include a dearth of mental health professionals, high treatment costs, long waiting lists specific to HICs and resistance to attending therapy due to stigma [96, 97]. Supporting this shift further, a few of these studies indicated that a supportive person consistently visiting the mother at home, providing positive feedback and lending an empathetic ear, is most likely, the process resulting in reduction in depression [26, 36, 42].

Taking the point on scarcity of mental health professionals further, our review found interventions delivered by non-specialist health workers performing better overall and surprisingly even for psychological approaches [53–55, 70, 73]. To further promote this notion of non-specialist delivery of interventions for PPD in terms of feasibility and effectiveness at a larger scale, two recent trials from India and Pakistan [53, 55], under the mother-focused psychological category, adopted a somewhat different strategy. They developed the THPP intervention with BA as their primary technique and a reduced emphasis on cognitive reframing, which was based on initial findings from their formative work wherein the delivery agents described cognitive restructuring as comparatively difficult to learn and apply. The intervention yielded moderate effects on PPD. Moreover, and somewhat expectedly, the psychosocial interventions seemed to rely heavily on non-specialist delivery and demonstrated positive PPD outcomes especially for mother-child focused interventions [36, 44, 46]. For mother-focused psychosocial interventions, however, only one [44] produced a positive impact of their intervention provided by peers. An exception to this was the task-sharing psychological intervention that was ineffective for treating PPD for which the authors put forward several probable reasons contributing to its non-success that have been explained above [48]. Backing our finding is the large trial from UK [13, 14] that demonstrated marked improvement in PPD amongst women treated by non-specialists as compared to those treated by specialists though they gave no clear indication regarding the substantial effectiveness of one delivery agent over the other. This could further depend upon the extent and quality of training and supervision provided to non-specialists [26]. These findings have important implications for development of inexpensive and ‘available at the doorstep’ interventions with a potential for scale-up.

One way of explaining the findings from our review is in terms of the role played by several factors in the change pathway of interventions. A promising study from Uganda [98] showed effects of a parenting intervention on maternal psychological well-being and child development vis-à-vis mediating factors from one year after childbirth. They ascertained perceived positive support as a mediator for maternal depressive symptoms and home stimulation as one for better child development outcomes. Further, they observed that the probability of PPD to have an impact on child’s mental development increased only in case of diminished responsive caregiving. The kind and severity of depression, it’s recurrent nature, time of onset–antenatal or postnatal, sample characteristics (high-risk, low-risk, with or without existing depressive disorders etc.), its naturalistic course of remission [99], life adversities [33, 100], social support and stress [15, 43], all are relevant for consideration while unpacking these pathways [24, 29, 101–103].

### Limitations

This review is subject to several limitations. First, we included only English language papers resulting in missing out on relevant studies that could have contributed to the depth and widened the scope of this review. Second, the quality of the studies varied. Third, we did not include any pilot studies, as we wanted to review evidence mainly on effectiveness and impact. An important aspect of this review is the inclusion of studies from both HICs and LMICs. More studies from the developed countries contributed to the review, once again reminding us about the glaring paucity of equivalent evidence from the developing world. However, the inclusion of both also in many ways strengthens the review by presenting a synthesis of findings from very diverse settings highlighting the similarities of challenges and ways in which PPD is being addressed across countries despite disparate social determinants.

### Conclusion

Our review provides strong evidence in favor of mother-focused interventions for addressing maternal PPD outcomes. A key highlight of the above synthesis is the comparatively higher effectiveness of mixed interventions that combine both psychological and psychosocial components in improving PPD outcomes. This could be attributed to the inclusion of an evidence-based psychological technique as a component of the intervention, along with providing the necessary social support, and having PPD as a primary focus. The last point is vital for expecting positive results for maternal PPD, regardless of which approach researchers wish to evaluate. A second key message arises from the comparison of mother-focused and mother-child-focused psychosocial interventions. It was fascinating to observe how their performance in improving PPD outcomes changed with the addition of child-related outcomes. This suggests that psychosocial interventions can be effective in reducing PPD symptoms once elements around the child’s outcomes are included in the intervention. However, more methodologically robust trials are required to ascertain the effectiveness of such interventions, especially since they aim to address both maternal PPD and child outcomes.

The review alerts us to important design elements for interventions to address the complexities around PPD. The first has to do with a clear focus: would the primary outcome of the intervention be PPD, any of the child outcomes, or the dyadic relationship? While narrowing conceptual focus, such clarity would also point to a suitable approach. For example, if the focus is to bring about a change in PPD, one might adapt a mixed approach—an evidence-based psychological technique such as CBT, BA or IPT, along with psychosocial support and probably individually delivered—to the intervention’s context. Alternatively, a focus on child outcomes might mean designing psychosocial strategies such as psychoeducation, responsive play and stimulation, and increased familial and social support. Secondly, identifying factors—e.g., duration of the treatment and follow-up, severity of PPD, how much at risk the target population is—that might have an effect on the uptake and impact of the intervention should be considered as the key principles of its design.

From the clinical and implementation perspective, two key points emerge from this review. First is the lack of apparent difference between specialists and non-specialists in producing positive changes in PPD. This is encouraging in terms of addressing ground-level realities of costs and availability. However, it is an area warranting further exploration as it has important policy implications. It could mean that training and supervising lay health workers to deliver such interventions might be vital to their sustenance, scalability and cost-effectiveness, especially in settings with minimal resources. The second key point is that interventions need to be delivered for a longer duration to bring about long-lasting or permanent change. Extended implementation managed by specialists has significant cost implications; trained non-specialists, on the other hand, might prove to be cost-effective in the longer run. These are questions that need further investigation from the perspective of researchers, decision-makers and, most importantly, mothers.

Finally, an astonishing finding of the review is the utter lack of evidence from LAMICs especially amongst mother-focused interventions. This highlights a huge gap, especially in evidence around interventions for maternal PPD. Further research is needed to fill this gap, and indeed to consider–In low and middle income settings above all—the many questions arising from the above review.

## Supporting information

S1 File Contains appendices. (DOCX)
