# Supplementary material for: Comparing the effectiveness of mother-focused interventions to that of mother-child focused interventions in improving maternal postpartum depression outcomes: A systematic review
Source: PLoS One. 2023 Dec 20;18(12):e0295955. doi: 10.1371/journal.pone.0295955 (PMC10732407; doi:10.1371/journal.pone.0295955)
Supplement: S1 File — (DOCX) [file pone.0295955.s001.docx]

# *Note:* These appendices are being submitted as the supplemental material along with the manuscript - “*Comparing the Effectiveness of Mother-Focused Interventions to that of Mother-Child Focused Interventions in Improving Maternal Postpartum Depression Outcomes: A Systematic Review*” These provide further details on the intervention content and results from each study included in the review.

# Appendix

## Appendix A: Systematic Literature Review Search Strategy

The strategy below was developed for MEDLINE database search. Similar strategy was applied across all the other databases.

**Table A.1: Search strategy developed for conducting database searches**

| **S. No.** | **Searches** | **Results** |
| --- | --- | --- |
|  | (Postnatal depress* disorder* or postpartum depress* disorder* or postnatal depress* symptom* or postpartum depress* symptom* or perinatal depress* or perinatal depress* disorder* or perinatal depress* symptom* or antenatal depress* disorder * or antenatal depress* or antenatal depress* symptom* or antepartum depress*z or antepartum depress* symptom* or antepartum depress* disorder* or maternal depress* or maternal depress* disorder* or maternal depress* symptom* or puerperal depress* or puerperal depress* disorder* or puerperal depress* symptom*).mp. [Mp=title, abstract, original title, name of substance word, subject heading word, keyword heading word, protocol supplementary concept word, rare disease supplementary concept word, unique identifier, synonyms] | 30920 |
|  | exp Postpartum Depression/ or maternal depression.mp. | 25242 |
|  | "Depression in pregnancy".mp. | 2709 |
|  | **1 or 2 or 3**  [4= all search term rows related to postpartum depression combined (#1, #2, #3)] | 34861 |
|  | limit 4 to yr="2003 - 2019" | 28977 |
|  | (Mother-child intervention* or mother-child intervention* or child health intervention or child growth intervention or child development intervention or maternal health intervention or "intervention in first 1000 days" or child care intervention or "maternal and child health intervention").mp. [mp=title, abstract, original title, name of substance word, subject heading word, keyword heading word, protocol supplementary concept word, rare disease supplementary concept word, unique identifier, synonyms] | 226 |
|  | limit 5 to yr="2003 - 2019" | 200 |
|  | **5 and 7**  [8 = search term rows for postpartum depression (#5) and mother-child interventions (#7) combined] | 30 |
|  | (psychological intervention* or psychosocial intervention* or mental health intervention* or CBT or Cognitive behavio?r therap* or cognitive behavioural?ral therap* or Cognitive behavio?r intervention* or Cognitive behavio?ral intervention* or behavio?ral activation or psychodynamic therap* or psychodynamic intervention*).mp. [mp=title, abstract, original title, name of substance word, subject heading word, keyword heading word, protocol supplementary concept word, rare disease supplementary concept word, unique identifier, synonyms] | 117185 |
|  | limit 7 to yr="2003 - 2019" | 97305 |
|  | **7 or 10**  [11 = search term rows for mother-child interventions (#7) and psychological or psychosocial interventions (#10) combined] | 20182 |
|  | (randomized controlled trial* or randomized controlled trial* or cluster randomized controlled trial* or cluster randomized controlled trial* or clinical trial* or evaluation study or evaluation studies or cross over study or cross over studies or controlled clinical trial* or cohort study or cohort studies or cross-sectional study).mp. [mp=title, abstract, original title, name of substance word, subject heading word, keyword heading word, protocol supplementary concept word, rare disease supplementary concept word, unique identifier, synonyms] | 4008840 |
|  | limit 12 to yr="2003 - 2019" | 2879472 |
|  | **5 and 11 and 13**  [14 = search term rows postpartum depression (#5) and psychological or psychosocial interventions (#11) and for randomized controlled trials (#14) combined] | 462 |
|  | Remove duplicates from 14 | 272 |

## Appendix B: Quality Assessment Checklist: Adapted From SIGN

| 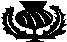 S I G N | | Methodology Checklist 2: Controlled Trials | | | | | | |
| --- | --- | --- | --- | --- | --- | --- | --- | --- |
| Study identification (*Include author, title, year of publication, journal title, pages*): | | | | | | | | |
| Guideline topic: | | | Key Question No: | | | Reviewer: | | |
| Before completing this checklist, consider:   1. Is the paper a randomized controlled trial or a controlled clinical trial? If in doubt, check the study design algorithm available from SIGN and make sure you have the correct checklist. If it is a controlled clinical trial questions 1.2, 1.3, and 1.4 are not relevant, and the study cannot be rated higher than 1+ 2. Is the paper relevant to key question? Analyze using PICO (Patient or Population Intervention Comparison Outcome). IF NO REJECT (give reason below). IF YES complete the checklist. | | | | | | | | |
| Reason for rejection: 1. Paper not relevant to key question □ 2. Other reason □ (please specify): | | | | | | | | |
| **Section 1: Internal validity** | | | | | | | |  |
| *In a well conducted RCT study…* | | | | | *Does this study do it?* | | |  |
| 1.1 | The study addresses an appropriate and clearly focused question. | | | | Yes □  Can’t say □ | | No □ |  |
| 1.2 | The assignment of subjects to treatment groups is randomised. | | | | Yes □  Can’t say □ | | No □ |  |
| 1.3 | An adequate concealment method is used. | | | | Yes □  Can’t say □ | | No □ |  |
| 1.4 | The design keeps subjects and investigators ‘blind’ about treatment allocation. | | | | Yes □  Can’t say □ | | No □ |  |
| 1.5 | The treatment and control groups are similar at the start of the trial. | | | | Yes □  Can’t say □ | | No □ |  |
| 1.6 | The only difference between groups is the treatment under investigation. | | | | Yes □  Can’t say □ | | No □ |  |
| 1.7 | All relevant outcomes are measured in a standard, valid and reliable way | | | | Yes □  Can’t say □ | | No □ |  |
| 1.8 | What percentage of the individuals or clusters recruited into each treatment arm of the study dropped out before the study was completed? | | | | 26.8% | | |  |
| 1.9 | All the subjects are analyzed in the groups to which they were randomly allocated (often referred to as intention to treat analysis). | | | | Yes □  Can’t say □ | | No □  Does not apply □ |  |
| 1.10 | Where the study is carried out at more than one site, results are comparable for all sites. | | | | Yes □  Can’t say □ | | No □  Does not apply □ |  |
| **Section 2: OVERALL ASSESSMENT OF THE STUDY** | | | | | | | |  |
| 2.1 | How well was the study done to minimize bias?  *Code as follows:* | | | High quality (++)□  Acceptable (+)□  Low quality (-)□  Unacceptable – reject 0 □ | | | |  |
| 2.2 | Taking into account clinical considerations, your evaluation of the methodology used, and the statistical power of the study, are you certain that the overall effect is due to the study intervention? | | |  | | | |  |
| 2.3 | Are the results of this study directly applicable to the patient group targeted by this guideline? | | |  | | | |  |
| 2.4 | Notes. Summarize the authors’ conclusions. Add any comments on your own assessment of the study, and the extent to which it answers your question and mention any areas of uncertainty raised above. | | | | | | |  |

## Appendix C: Outcome Measures - Maternal PPD and Child Outcomes Across Studies Reviewed

Sixteen different scales and/or checklists were used to measure maternal depression and severity of symptoms listed in the table below. The outcomes were assessed at various time points ranging from 2^nd^ trimester to 5 years after childbirth.

**Table C.1: List of outcome measures for assessing Depression used across studies**

| **S. No.** | **Outcome measures used in the studies reviewed** | **No. Of studies using each measure** |
| --- | --- | --- |
|  | Edinburgh Postnatal Depression Scale (EPDS) | 23 |
|  | Structured Clinical Interview for DSM-IV Axis 1 Disorders (SCID-I) | 10 |
|  | Beck Depression Inventory (BDI) | 9 |
|  | Patient Health Questionnaire (PHQ-9) | 4 |
|  | Hamilton Depression Rating Scale (HDRS) | 4 |
|  | Depression Anxiety Stress Scales (DASS) | 3 |
|  | Behavioral Activation for Depression Scale – Short Form (BADS-SF) | 2 |
|  | Centre for Epidemiological Studies Depression Scale (CES-D) | 2 |
|  | Mini International Neuropsychiatric Interview (MINI) | 2 |
|  | World Health Organization Disability Assessment Schedule (WHODAS) 2.0 | 2 |
|  | General Health Questionnaire (GHQ) | 1 |
|  | Mood Screener | 1 |
|  | Leverton Questionnaire (LQ) | 1 |
|  | Kessler 10 (K10) | 1 |
|  | Montgomery–Åsberg Depression Rating Scale – Self rated (MADRS-S) | 1 |
|  | Cape Town Functional Assessment Instrument for Perinatal depression (FAI) | 1 |
|  | Postpartum Depression Screening Scale, Short Form (PDSS-SF) | 1 |
|  | Composite International Diagnostic Interview (CIDI) | 1 |

**Table C.2: List of outcome measures for assessing child health outcomes used across studies**

| **S. No.** | **Outcome measures used in the studies reviewed** | **No. Of studies using each measure** |
| --- | --- | --- |
|  | Standard child anthropometry measures [length (or height) and weight] | **3** |
|  | Bayley Scales of Infant and Toddler Development-Third Edition (BSID-III) | **2** |
|  | Griffiths Mental Development Scale [Developmental quotient (DQ)] | **1** |
|  | Specially designed questionnaire for diarrhoeal episodes | **1** |
|  | Immunisation Records at 12 months of child’s age | **1** |
|  | 10 minutes video tapes of free play for quality of mother-infant interactions | **1** |
|  | Ainsworth Strange Situation procedure | **1** |
|  | Neonatal Mortality Rate: Key informant surveillance system & verbal autopsies | **1** |
|  | Behavioural Screening Questionnaire (BSQ) | **1** |
|  | Child Behaviour Checklist (CBCL) questionnaire | **1** |
|  | Attachment Q-Sort (AQS) | **1** |
|  | Early childhood behaviour questionnaire (ECBQ) - Parent report | **1** |
|  | Laboratory Temperament Assessment Battery (Lab-TAB) | **1** |
|  | Mental Development Index of the Bayley Scales of Infant Development | **1** |
|  | The McCarthy Scales of Children’s Ability | **1** |
|  | Pre-school Behaviour Checklist (PBCL) [Teacher’s report) | **1** |
|  | Rutter A^2^ Scale (Mother’s self report) | **1** |
|  | Home Inventory for Measurement of the Environment - Infant  Toddler version (HOME-IT) | **1** |

## Appendix D: Characteristics of the Interventions

For this review, we divided the different kinds of intervention delivery agents into three categories: (a) Health professionals, (b) paraprofessionals, and (c) community volunteers. In eight studies, the comparison group received additional inputs besides TAU including extra information on PPD, provision of information materials or access to Internet sites on depression, and permission to take treatments based on participant's own choice. In a couple of studies, the comparison group was another treatment.

Note: The content of the treatments has been directly extracted from the published articles.

**Table D.1: Characteristics of the Intervention: Mother-focused Interventions**

| **S. No.** | **Source** | **Components of intervention: Mother-focused** | | | | **Comparison group** | |
| --- | --- | --- | --- | --- | --- | --- | --- |
|  |  | 1. **Type** 2. **Mode of Delivery** 3. **Duration** | **Content** | **Delivered by** | |  | |
|  | **Psychological** | | | | | | |
|  | Quasi Experimental  **Huynh-Nhu Le (2021)** | 1. CBT 2. Group 3. Six weekly sessions | - “Six sessions included three modules teaching women about the relationship between mood and: (a) thoughts, (b) activities, and (c) relationships with others, consistent with a cognitive-behavioural and attachment theory and adapted to the contextual factors of low-income Latinas. The intervention was taught in Spanish.” | - (1)Health professionals: mental health professional supervised by first author - (2) Para professionals: post- bachelor’s trained bilingual and bicultural research staff.” | | No comparison group. Before after study design. | |
|  | RCT  **Jannati 2020** | 1. CBT 2. Individual | - “Developed a mobile application called "Happy Mom” – based on CBT principles. - Comprised eight lessons conducted over eight weeks. - Session 1: Introduction and goal setting. Session 2: Getting activated: mothers set goals to work towards. Session 3: Emotional recognition: mothers see the link between their thoughts, feelings and behaviour. Session 4: Noticing thoughts: mothers learn about thoughts getting distorted when depressed. Session 5: Thought challenging: mothers learn how to challenge and modify. Session 6: Problem-solving. Session 7: Improving social skills - learning interpersonal engagement, building, and maintaining friendships. Session 8: Relapse prevention: mothers set future goals and prepare personal action plan.” | - Mobile application - Content developed by psychologists | |  | |
|  | Cluster RCT  **(Ngai 2019)** | 1. CBI 2. Individual & Group (antenatal session conducted in groups of 10 pairs of couples) 3. “One 3-hour antenatal group session and two structured 30- minute follow-up sessions on the telephone conducted separately with each parent at 2 and 4 weeks postpartum by the same midwife. ” | - A structured protocol was developed - “Sessions covered: (1) an overview of stressors in postnatal period, signs and symptoms of PND; (2) cognitive restructuring techniques; (3) problem-solving, goal-setting, and decision- making skills to manage common neonatal problems; and (4) communication skills training to improve interpersonal relationships. - Prenatal care was offered to women early in gestation at antenatal clinics and subsequent visits to the hospitals or maternal and child health clinics. Women could obtain information on the process of pregnancy, childbirth, and basic childcare skills by attending the routine antenatal classes organised by the hospitals or maternal and child health clinics. - All participants received TAU - prenatal and postnatal care as prescribed in Hong Kong - women were offered a 6-week postpartum follow up at the hospitals or maternal and child health clinics. | - Paraprofessionals: Experienced midwife with prior experience in conducting cognitive behavioural interventions for PND; received 20 hours’ training in CBT by second author, a clinical psychologist and . weekly supervision by research team. | | - Same CBI delivered to mothers alone in the comparison group by the same midwife. - All participants received TAU - prenatal and postnatal care as prescribed in Hong Kong. | |
|  | Cluster RCT  **Gureje (2019)** | 1. PST 2. Individual 3. “(1) 8 weekly antepartum sessions. (2) Depending on EPDS scores (<12 or **≥**12), either four fortnightly top-up sessions or eight weekly sessions. Commenced 6 weeks post-delivery. (3) For participants still with EPDS scores **≥**12: reassessed by community physician to initiate pharmacotherapy plus psychological intervention (each session approx. 30**–**45 min) or specialist referral.” | - “High-intensity treatment (HIT) plus EUC, a stepped-care treatment, using manualised psychological intervention package. - Core component – locally adapted PST for primary care. Patient guided through a step-by-step process of breaking down current psychosocial stressors and exploring solutions, including using personal resources and available social support. - Mothers received parenting skills training; information on importance of routine antenatal visits; adequate nutrition and rest; newborn care and nutrition; responsive stimulation with infant. - First session: Familiarizing and psychoeducation. Information booklets in local language provided. Second session: Parenting skills and activity scheduling (BA). Third session: PST. Seventh session: patient exploring social network, select most appropriate supportive person for childcare and household chores post-delivery. Last antenatal session: reviewed patients**’** progress and emphasized using skills acquired. Post-delivery sessions: revisited issues considering arrival of new baby, explored further issues of social (& marital) support, & parenting skills.” | Paraprofessionals: government frontline workers called the Primary Maternal Care Providers (PMCP) | | “EUC or Low intensity treatment (LIT):   - Specifications of the WHO mhGAP-IG as adapted for Nigerian health system. - PMCP delivered intervention: psychoeducation, addressing current psychosocial stressors, reactivating social network. No structured sessions stipulated, no stepped-care procedure specified; number/frequency and content were at the discretion of PMCP” | |
|  | RCT  **Lund (2019)** | 1. Task-sharing multicomponent psychological 2. Individual 3. Six weekly sessions (45-60 min) | - “Structured, manualised psychological treatment. Six counseling sessions adapted: psycho-education, problem solving, BA, healthy thinking, relaxation training, and birth preparation to help build resilience and social support for women experiencing low mood in the context of social and interpersonal adversity in Khayelitsha. Manual included colloquial idioms for distress identified during formative research. - At each session, participants' health and suicidal risk assessed using a checklist.” | Paraprofessionals:   - Six Community health workers (CHWs) recruited from a local NGO - Two CHWs recruited from another NGO trained to conduct the phone calls in control arm, but not trained in counseling techniques used in the intervention arm | | “EUC: Monthly phone calls for three months, in addition to routine antenatal health care provided by the clinic. A set protocol followed using a checklist including items on participant's health, major life changes, mental health support received, and experience of depressive symptoms or suicidal ideation.” | |
|  | RCT  **Fuhr (2019)** | 1. BA 2. Individual 3. A minimum of 6 to a maximum of 14 sessions delivered in four phases over 7–12 months, each session 30-45 min | - BA + EUC - “THPP – more focus on BA and less on cognitive restructuring. Health charts with pictorial illustrations aimed to improve mother**’**s personal health, social support and bonding with her infant; used to set goals and to monitor mother**’**s healthy activities. Most activities revolved around child-care, family support for these activities so the mother could enjoy them and find time for self-care. Health messages standardized for lay peer volunteers. - Peers to refer women with general health needs to Anganwadi workers. - Culturally appropriate illustrations expanded into vignettes with CBT-based narratives for delivery through **‘**story-telling**’,** depicting different moods, behaviour and thoughts to encourage participants identify links between unhelpful behaviour, thoughts and depressed mood.” | Community volunteers: Peer counselors   - Laywomen (i.e., without any mental health training) with an interest or desire to help and support other women within their community - Middle-aged with children - With a similar socio-demographic background as the participants - Selected for their good communication skills - Referred to as Sakhi, which translates to friend in Hindi | | “EUC alone:  Standard care from gynecologist and enhanced treatment, patients and gynecologists informed if participant screened positive for depression; gynecologists given the adapted WHO mhGAP-IG for perinatal depression, with information on patient referral for severe depression suicide risk to specialist mental health-care facilities; participants provided with an information sheet with details on accessing appropriate health care during pregnancy and beyond.” | |
|  | RCT  **Sikander (2019)** | 1. BA 2. Individual + Group 3. A maximum of 10 individual sessions (four during prenatal period and six during postnatal). Additionally, four group sessions were integrated into routine monthly **‘**women**’**s groups**’** organized by LHWs for all perinatal women (including non-depressed women) | - BA + EUC - THPP as described above in point no. 3 - “Peers to refer women with general health needs to LHWs. - Peers delivered groups in partnership with LHWs, and included general psychoeducation and child development information. Peers motivated depressed mothers to attend groups in order to reactivate their social networks.” | Community volunteers: Volunteer peers (worked closely with government LHWs)   - Local married women from community with no prior health training or experience - 30-35 years of age with children - Shared socio-demographic and life experiences with target population - Selected for good communication skills - Referred to as *Razakars* (roughly translates to “volunteer helpers” in local language Urdu). | | “EUC alone: Standard care from LHWs. Treatment enhanced in following ways: (1) participants and LHWs informed of screening results (2) doctors and midwives at primary health centers given adapted mhGAP-IG.  Rest is same as point 3 above.” | |
|  | RCT **Dimidjan (2017)** | 1. Structured BA 2. Individual; 3. 10-session protocol with flexibility regarding spacing and number of sessions to accommodate pregnant women’s scheduling demands. | “Treatment included case conceptualization and formulation of treatment plan. Key treatment strategies: self-monitoring, scheduling activities, problem solving, increasing social support and interpersonal communication skills. Between-session homework. Clinical examples and assignments tailored to circumstances and challenges of pregnancy & early postpartum.” | (1) Health professionals: Nurse practitioner and  registered occupational therapist.  (2) Paraprofessional: midwife | | “Routine care and completing study assessments. In case of increased depression symptom severity, participant and obstetric provide notified and referrals made to the behavioural health provider at treatment site.” | |
|  | RCT **Forsell (2017)** | 1. CBT 2. Individual self-help; 3. 10 weeks as an add-on to maternity care. | “ICBT - guided self-help treatment; reading material (about 75,000 words), assessments, homework and work sheets delivered via secure online platform. CBT-trained supervised therapist provided regular feedback, encouragements and support in written messages. Adapted version of ICBT currently in use in regular care at the Internet Psychiatry Clinic in Stockholm since 2007. Content included description of depression and its relation to pregnancy; BA, cognitive restructuring (sense making, de-stigmatization, contrast between societal norms and social expectations about how one ‘should’ feel and think when pregnant rather than how usually many women feel), relationships and role transitions, anxiety and worry, and sleep problems.” | (1) Self  (2) Health professionals: Doctors, psychologists, psychiatrists and obstetrics for regular supervision and feedback | | “Continuation of their current maternity care for 10 weeks, followed by optional ICBT.” | |
|  | RCT **Pugh (2016)** | 1. CBT 2. Individual self-help; 3. Seven online modules. Participants encouraged covering one module/week | “TA-ICBT had seven modules including a range of media (e.g., text, graphics, animation, audio, video). Each page viewed prior to proceeding to the next page. Check-in questions focusing on module content presented at beginning while homework exercises assigned at the end. Therapists emailed their assigned participant on a set day each week for support, encouragement, and to answer questions. Composing e-mails took 15 to 20 minutes on an average and were not pre-prepared. Content was individualized. Therapist to telephone the participant in case of significant distress or failure to log on for over seven days.” | Health professionals: Clinical Psychology doctoral students as internet therapists; registered psychologist and expert in TA-ICBT as supervisor for internet therapists. | | “Waitlist Control: Participants given information pamphlet on PPD and websites to access provincial mental health support services. Participants to inform researchers of any treatment received during wait period.” | |
|  | RCT **Milgrom (2016)** | 1. CBT 2. Individual: mostly self-help; 3. 6 weeks (one session/week) 4. Mother-focused | “Web based content presented using text, animations, video introductions, case vignettes, and audio and video tutorials. Six sequential sessions made available weekly: (1) Getting Started, (2) Managing Mood, (3) Increasing Pleasant Activities, (4) Managing Negative Thoughts, (5) Increasing Positive Thoughts, and (6) Planning for the Future. Participants enter own mood and activity data through self-monitoring tools; enabled daily tracking and online charting.” | (1) Health professionals: clinical and health psychologist (2) Paraprofessionals: NGO workers: graduate psychology trainees, supported and supervised by 2 senior psychologists. (3) Self | | “TAU varied at the discretion of each participant’s nominated health professional, expected to include heterogeneous mix of interventions. Participants given links to general Internet resources on mental health. Received email prompts to complete online assessments.” | |
|  | RCT **Jesse (2015)** | 1. CBT 2. Group (Total 21 groups with 2 to 6 women); 3. 6 weeks; 2 hours, once a week. | “Culturally tailored manualised CBI for rural and minority pregnant low-income women. (3a) MP3 player with pre-programmed play list of weekly homework review; stress reducing guided visualization; review of thoughts, feelings, and behaviours; positive affirmations; motivational and inspiring music. Women recorded positive affirmations on MP3 player for later listening. (3b) Facilitator’s manual included organization, format, flow of activities for each group session.” | (1) Health professionals: licensed clinical social worker, licensed mental health professionals (2) Paraprofessional: NGO worker: resource-mom, staff member from community holding Associate Degree in Human Service Technology | | “(1) LHD: prenatal care from certified nurse-midwives (CNMs) or from a women’s health nurse practitioner (WHNP) (2) Regional perinatal centre: prenatal care from WHNP, obstetrical residents, or physicians.  (3) Both sites: regularly scheduled child birth education classes.” | |
|  | Quasi experimental study: pre‑ and post‑test design **Ashtiani (2015)** | 1. CBT 2. Individual 3. 8, 40–60 min sessions of CBT intervention integrated into women’s standard prenatal visits | “Delivered by recorded film and interactive workbook. ECBSP included identifying & testing automatic negative thoughts, training in self‑monitoring, self‑focused attention, relaxation, thought restructuring, behavioural tasks, exposure to worry cues. Other themes included education on depression and anxiety, positive communication, realistic expectation about pregnancy, delivery, and parenting. Homework to ensure skills transfer.” | (1) Health professionals: clinical psychologist (2) Paraprofessional: midwives | | “Routine antenatal care by obstetrics nurses.” | |
|  | Quasi Experiment **Dimidjan (2014)** | 1. MBCT 2. Group 3. Ten series of classes; each series included eight 2-hours sessions delivered in clinics. | “Adapted for perinatal women (MBCT-PD): includes increased attention to brief informal mindfulness practices (e.g., washing dishes and driving), customized mindfulness and yoga practices (e.g., “being with baby” informal practice, and prenatal yoga poses), and psychoeducation on perinatal depression, parenthood, self compassion, self-care, social support. Audio-recorded files provided each week to guide home practice and a DVD to guide yoga practice.” | Health professionals: Clinical psychologists | | No comparison group. | |
|  | RCT **Tandon (2014)** | 1. CBT 2. Group; 3. 6 weekly sessions consisting of six two-hour. | “Standard home visiting plus adapted version of the Mothers and Babies Course (MB Course). Sessions divided into three two-session modules mapping onto core CBT concepts. Each session contained didactic instruction on core content, activities and group discussion on introducing and practicing the use of core skills (e.g., strategies to reduce harmful thought patterns, ways to effectively ask for support). A personal project assigned at end of each session encouraging practice of skills taught. Booster sessions conducted at 3-months and 6-months post-intervention.” | Health professionals: licensed clinical social worker or clinical psychologist. | | “All women received standard home visiting services plus a packet of information on perinatal depression”. | |
|  | RCT **Jiang (2014)** | 1. CBT 2. Individual; 3. Face-to-face counseling: 40 min session once a week; telephone counseling: once a week. | “Specific psychological interventions included: (a) Health education: mailing PPD prevention and treatment manuals; CD on pathogenesis, risk factors, PPD prevention focusing on solutions to social support, marital relationship, child-rearing responsibilities for families. (b) Out-patient counseling: face-to-face, personalized psychological counseling on cognitive errors, establish self-esteem, recommend reasonable solution to societal and family problems (c) Telephone counseling: inquiry hotline set up and a special person designated, calls made to those unable to come to the hospital (d) Referral: those with severe PPD clinical symptoms after intervention transferred to psychiatric specialized hospital for further treatment.” | Paraprofessionals: Frontline workers (medical staff) | | “Conventional method: Medical staff informed women to come to medical institutions for further consultation and medical treatment.” | |
|  | Quasi experimental study **Hou (2014)** | 1. CBT + Systemic Family Therapy 2. CBT in individual format and SFT in group with one family; 3. 3 months: Sessions began 2 months post- and discontinued 5 months post-delivery. CBT: 13 weekly sessions (60 min).  SFT: 6 twice-monthly therapeutic interviews (60-90 min). CBT alternated with SFT. | “(1a) CBT: Stage 1: Therapeutic alliance, determining therapeutic goals. Stage 2: cognitive restructuring. Stage 3: Role-play; training of recognition behaviours. Psycho-education, case analysis, and homework constituted these 3 stages. Stage 4 (ending counseling): Approve efforts and progress; help deal with closure; remind applying techniques in daily life.  (1b) SFT: (i) Circular, feed-forward, differentiated, hypothetical questioning to understand family factors affecting patients (ii) introduce new perspectives & measures to alter illness related feedback loop, reconstruct interaction mode among family members, (iii) homework on role reversal, regular communication to help members create & consolidate good family rule and mode of interaction.” | (1) Paraprofessionals: NGO workers: received systematic training for CBT and SFT  (2) Health professionals: Experienced psychologists did evaluations | | “Routine postnatal care”: not detailed. | |
|  | RCT **Pinheiro (2013)** | 1. CBT and RCT 2. Individual; 3. Not mentioned. | “CBT manual included session script to determine patients mood, bridge with previous session, establishing session's agenda, homework review, setting up household tasks, final review, and feedback.” | (1) Health professionals: Clinical psychology interns trained by study coordinators (psychologists and psychiatrists with training in CBT and RCT). | | “RCT: same number of sessions as CBT. Manual included session script covering interpreting new situations, allowing patient to recover the lost feelings of intelligibility and transformation.” | |
|  | RCT **Ammerman (2013)** | 1. CBT 2. Individual; 3. 15 weekly 60 minutes sessions, plus a booster session 1-month post treatment. | “Two home visitation models used: Nurse-Family Partnership (NFP) and Healthy Families America (HFA). Mothers enrolled prior to 28 weeks gestation in NFP and from 20 weeks gestation to 3 months of child age for HFA. Focus and content followed CBT directives, included BA, cognitive restructuring and relapse prevention. Mothers not permitted to receive additional treatment during trial’s treatment phase. Goals: (a) improve pregnancy outcomes through nutrition education and substance use reduction (b) support parents in providing children with safe, nurturing, stimulating home environment (c) optimize child health and development (d) link families to health care and other needed services (e) promote economic self-sufficiency.” | Health professionals: IH-CBT delivered by licensed master's-level social workers. Weekly supervision by doctoral-level clinicians. | | “Standard home visitation (SHV): mothers received regular home visits as per HFA and NFP model, emphasizing child health and development, nurturing mother-child relationship, maternal health & self sufficiency, linkage to other community services. Mothers permitted to receive treatment for depression in the community.” | |
|  | RCT **O'Mahen (2013) +1** | 1. BA 2. Individual; 3. 11 weekly sessions, up to 40mins each. | “Treatment adapted for postnatal online delivery from BA manual. Multimedia in presentation. Weekly e-mail reminders sent. Access to an iBA-specific online chat room moderated by parent supporter and specialist health visitors.” | (1) Health professionals: specialist health visitors (2) Community volunteers: parent supporters (3) Paraprofessional: Mental health workers with undergraduate degrees and 1 year clinical qualification in psychological therapies (UK Improving Access to Psychological Therapies (IAPT) training scheme). | | “The TAU condition was allowed to vary as per usual practice. Women in both groups had access to Netmums' general depression chat room throughout the study.” | |
|  | RCT **Milgrom (2011)** | 1. CBT: Counseling 2. Individual; 3. Six weekly sessions of manualised Adjunctive counseling-CBT; 3 fortnightly GP appointments in all groups. | “Three study groups: (a) Group A: GP management (trained in PND management) (b) “Group B: Adjunctive counseling-CBT delivered by trained nurse at maternal child health centers (c) Group C: Adjunctive counseling-CBT delivered by experienced psychologist at hospital Psychology dept. (b and c adjunct to GP management). Involved screening, diagnosis with DSM-IV, risk assessment & management, engagement, bio psychosocial model of PND, medication during lactation, common patient concerns, referral, principles of treatment (supportive counseling & CBT strategies). Covered 3 phases: assessment, goal setting, and treatment. Sessions focused on PPD psycho-education, goal setting, problem solving, and behavioural and basic cognitive techniques. Add on: partner relationship, social support, and mother-baby relationship.” | Health professionals: Doctors (woman’s GP), Nurses, psychologist | | “Management by GP (trained in PND management) alone and 3 “Fortnightly GP appointments.” | |
|  | RCT **Le (2011)** | 1. CBT 2. Group and individual; 3. 8 weekly 2-hours group intervention during pregnancy and 3 individual booster sessions (6 weeks, 4 and 12 months postpartum). | “The MB course taught in Spanish and consisted of psychoeducational group sessions, teaching women mood regulation skills to prevent perinatal depression. Included detailed instructor and participants’ manuals. Individual booster sessions to review main concepts and to generalize techniques to the role as new mothers.” | Paraprofessionals: NGO workers: post bachelor’s trained bilingual and/or bicultural research staff with training and weekly supervision. | | Usual care: not detailed. | |
|  | Cluster RCT **Brugha (2010)** | 1. CBT or person centred 2. Individual; 3. 8 weekly one-hour visits (maximum 8 weeks), commencing around 8 weeks postpartum. | “The HV-provided psychologically informed sessions were offered to women who had EPDS score >12 at 6 weeks (postal) and 8 weeks (face-to-face) postpartum. They were trained in one of two distinct psychologically informed approaches [a cognitive-behavioural approach (CBA) and a person-centred approach (PCA)], with additional supervisory support.” | Health professionals: Specialist Community Nurse (called Health Visitor (HV)) | | Postnatal Care as usual (CAU): not detailed. | |
|  | RCT **Austin (2008)** | 1. CBT 2. Group; 3. 6 weeks; 2-hour weekly sessions (and a later follow-up session). | “(1) CBT intervention manualised, focusing on preventing and managing stress, anxiety and low mood in pregnancy and newborn care. (2) Components: education about perinatal anxiety and depression, infant needs, behaviour in the first few months of life, pleasant event scheduling, relaxation training, goal setting, problem solving, cognitive reframing, assertion skills, broadening social support network, including local postnatal services.” | (1) Health professionals: clinical psychologist. (2) Paraprofessional: specifically trained midwife as co-therapist. | | “Information booklet alone: Women given a booklet containing information regarding risk factors for postnatal anxiety and depression, triggers for postnatal distress, strategies to prevent and/or manage such problems, a list of local postnatal support services, and accessing them.” | |
|  | **Psychosocial** | | | | | | |
|  | RCT **Lara (2010)** | 1. Psychoeducational strategies + non-directive counseling + supportive interactions 2. Group 3. Eight two-hour weekly sessions ≤ 15 participants per group. | “Two manuals (facilitator's and participant's): information, check lists and working forms with three components: (a) educational, acknowledgement and discussion of “normal” perinatal period and risk factors; (b) psychological, strategies to reduce depressive levels (e.g., increasing positive thinking and pleasant activities, improving self-esteem, etc.); and (c) group component involving creation of a trusting and supportive atmosphere.” | Paraprofessional: NGO Workers: with 5-25 years clinical experience | | “Usual care provided by their institution: both groups received copies of a self-help book on depression.” | |
|  | RCT **Dennis (2009)** | 1. Supportive interactions via peer support 2. Individual telephone based 3. A minimum of four contacts and then as required. Telephone contact initiated 48-72 hours after randomization. | - Peer support + usual care - “Peer support: specific type of social support incorporating informational, appraisal, and emotional assistance. Provided by lay volunteers, not from mother’s family or immediate social network, possessing experiential knowledge of PPD, sharing similar demographic characteristics. A mother-to-mother telephone-based support intervention developed: ‘Mothers Helping Mothers with PPD’.” - “Peer volunteers trained in a 4-hour session, provided with a take home 121 page manual for guidance and outlining professional referral services. Topics included peer support definition, potential benefits, relationship building, skills and techniques for effective telephone support, general PPD information, and helping process.” | Community volunteers: Peer volunteers; women from the community with a self-reported history of and recovery from PPD | | Usual care: “Women could access standard community postpartum care, including, services from public health nurses, physicians, other providers, and various community resources, including drop-in centers.” | |
|  | RCT **Ho (2009)** | 1. Psychoeducational strategies 2. Group 3. Unclear (One group session on 2nd day after delivery). | “Three-page booklet containing incidence, symptoms, causes, and management information about PPD. Women received this plus discussed it with primary care nurses on the second day post-delivery.” | Health professionals: Postpartum ward nurses | | “General postpartum education.” | |
|  | **Mixed** | | | | | | |
|  | RCT **Gao (2015)** | 1. IPT + Psychoeducation 2. Individual: face-to-face and telephone follow up; 3. One 1-hour education session before discharge and one telephone follow-up within the 2 weeks after discharge from the hospital. | “Specific IPT techniques, such as, information giving, use of affect, clarification, signaling what is significant, reviewing relationship and communication patterns, and providing social support. After the session, the new mother was given the written material for the programme. Protocol from previous study (Gao et al., 2010a) adapted to the individual context was used for education session.” | | Paraprofessional: Midwife educator with experience in delivering IPT oriented intervention | | “Standard care consisting of childbirth education: Visit from a nurse in the postnatal ward, distributing pamphlet on sources of assistance for mothers regarding hospital discharge” |
|  | RCT **Kozinsky (2012)** | 1. Group psychotherapy for PPD + IPT + CBT elements + psycho-educational strategies 2. Group; 3. Four 3-hour sessions for 4 consecutive weeks from the 25th week of gestation. | “Using information giving, interpersonal, group and CBT components, assertiveness and relaxation training.” | | (1) Health professionals: Doctors: Psychiatrists; (2) Paraprofessional: NGO workers: health visitors with training in Psychiatry | | “4 sessions providing routine education on pregnancy, childbirth, and baby care similar to usual care.” |
|  | RCT **Gao (2011)** | 1. IPT + Routine antenatal education 2. Group sessions 3. (1) Two 90-min sessions and one telephone follow up within 2 weeks after delivery. | “Routine antenatal education + IPT. (3a) First session: transition to motherhood, communication skills, baby gender issues and PPD information. (3b) Second session: developing social support, post-delivery interpersonal conflict resolution skills, issues regarding Chinese postpartum practice "doing the month". Used specific IPT techniques - information giving, clarification, communication analysis, role-playing and brainstorming. Written material provided. Telephone follow-up aimed to reinforce skills learned and address current or anticipated mood changes associated with interpersonal difficulties.” | | Paraprofessional: midwife educator trained in IPT oriented intervention | | “Routine antenatal classes in the study venue. Two 90-min sessions conducted by midwives. Content focused on providing information about labor and basic baby care skills with lecture and video as the main teaching methods.” |

**Table D.2: Intervention Characteristics and Content Analysis: Mother-Child Focused Interventions**

|  | **Source** | **Components of intervention: Mother-child focused** | | | **Comparison group** |
| --- | --- | --- | --- | --- | --- |
|  |  | 1. **Type** 2. **Mode of Delivery** 3. **Duration** | **Content** | **Delivered by** |  |
|  | Psychological | | | | |
|  | Cluster RCT **Rahman (2008)** | 1. CBT 2. Individual 3. Total 16 sessions: 4 weekly in last month of pregnancy - 3 sessions in first postnatal month, and 9 one-monthly sessions. | “Thinking healthy manual: - manualised CBT techniques of active listening, collaboration with the family, guided discovery (i.e., style of questioning to both gently probe for family’s health beliefs and to stimulate alternative ideas), and homework (i.e., trying things out between sessions, putting what has been learned into practice).” | Paraprofessional: Govt. Frontline workers: Lady Health Worker | “Equal number of home visits in the same way as those in the intervention group, but by routinely trained Lady Health Workers.” |
|  | **Psychosocial** | | | | |
|  | Cluster RCT **Tripathy (2010)** | 1. Supportive interactions via participatory group meetings 2. Group and individual; 3. Monthly group meeting (total 20). One facilitator covered 13 different groups every month. | “Participatory action and learning for women: Information on clean delivery and care seeking shared through stories and games, not as key messages. Picture-card games, role-play, and story-telling methods used to discuss cause-effect of typical problems in mothers and infants, devised prevention strategies, homecare support, and consultations. Facilitation materials adapted from Makwanpur, Nepal study.” | Community volunteers: Local woman; identified by community | “Enhanced care with formation of cluster level committees.” |
|  | RCT **Cooper (2009)** | 1. Psychoeducational strategies + counseling support via home visits 2. Individual; 3. A total of 16 visits ending at 5 months of child's age: Hour-long home visits made twice antenatal, weekly postpartum for 8 weeks, fortnightly for next 2 months, and monthly for another 2 months. | “Support plus ‘The Social Baby’ principles. WHO’s Improving the Psychosocial Development of Children programme principles incorporated in the adaptation of the Health Visitor Intervention Programme? Neonatal Behavioural Assessment Schedule (NBAS) to sensitize mothers to their infants’ abilities and needs.” | Paraprofessional: NGO worker: women, resident, selected with help from local community council. | “Standard health care: Fortnightly home visit from a community health worker assessing maternal and infant health, encouraging mothers for infant immunization and weight checks.” |
|  | Cluster RCT **Hennigham (2005)** | 1. Psychoeducational strategies + supportive interactions 2. Individual 3. Weekly one-hour home visit for one year. | “Homemade materials used to demonstrate play activities to mothers, parenting issues discussed. Visitors trained to ensure that both mothers and children experience success and feel competent. Parenting issues included importance of praise, attention, responsiveness, appropriate discipline strategies, child nutrition, promoting children’s play and learning. Other caregivers (fathers, grandparents) were encouraged to participate. No counseling or problem solving explicitly included in the intervention.” | Paraprofessionals: Frontline worker: Community health aides - employed in government health centers. | “Standard health and nutrition care: Govt. health workers provided routine care but not material on intervention.” |
|  | **Mixed** | | | | |
|  | RCT  **Stein (2018)** | 1. CBT + parenting video-feedback therapy (VFT) VS. CBT + progressive muscle relaxation (PMR) 2. Individual face to face sessions 3. Six weekly sessions and five fortnightly sessions (total 11 home-based sessions) before child age 1 year, followed by two booster sessions (between 6 months and 10 months) after the end of therapy in the second year | 1. “First session was CBT; second was either VFT or PMR. All subsequent sessions equally divided between CBT and either VFT or PMR (45 min each). 2. VFT aimed to improve quality of the mother–child interaction by enhancing three core parenting skills: maternal attention to infant cues and associated contingent responsiveness; emotional scaffolding; sensitivity and treating the child as a psychological agent, particularly in the context of attachment needs. 3. CBT targeted symptoms of depression. BA was the principal focus of intervention because it allowed directing therapeutic efforts to features common in PPD, including withdrawal and absence of routine. E.g., intervention was adapted to management of sleep routines, self-care activities, and support networks. Cognitive techniques included in latter sessions.” | Professionals: Qualified clinical psychologists, all with specialist CBT training | “ CBT + PMR:   1. Exercises in tensing and relaxing major muscle groups combined with attention to sensations. Participants given an audio recording of 16 pre-recorded tracks comprising guided relaxation exercise to enable practice between sessions. CBT same as intervention group.” |
|  | RCT **Husain (2017)** | 1. CBT + non-directive counseling + supportive interactions 2. Group; 3. 10 sessions delivered weekly in 60–90 minutes over 12 weeks. | “LTP Plus has two components – (a) Learning Through Play (LTP): developmentally & culturally appropriate messages to stimulate early child development. Pictorial calendar depicting eight successive stages of child development from birth to 3 years, illustrations of parent-child play and other activities to promote parental involvement, learning, and attachment. (b) THP: manualised, step-by-step instructions for 5 modules focusing on three areas: mother’s personal health, mother-infant relationship, & psychosocial support of significant others. “Here and now” problem-solving approach using CBT techniques of active listening, changing negative thinking, collaboration with family, guided discovery, homework, education on symptoms of depression, management, social support, practical advice on using healthcare.” | Health professionals: Graduate psychologists with limited clinical experience; supervised regularly by a senior clinical psychologist. | “Routine follow-ups by lady health workers (LHW) or traditional birth attendants through monthly home visits. LHW covers maternal, newborn and child care, family planning support, immunization.” |
|  | RCT **Cooper & Murray (2003)** | 1. CBT + Psychodynamic therapy + non-directive supportive counseling 2. Individual; 3. 10 weekly sessions from 8 to 18 weeks post-partum. | “(a) CBT: directed at problems identified by mother in infant management and observed problems in quality of mother–infant interaction; using problem solving, examining thinking patterns, modeling & reinforcement. (b) Psychodynamic therapy: exploring mother’s own early attachment history to promote representation of her infant and their relationship.  (c) Non-directive counseling: women given opportunity to share feelings about current concerns e.g., marital or financial, as well as concerns about their infant.” | (1) Health professionals: Specialists (2) Paraprofessional: NGO workers: non-specialist health visitors trained in two treatments | “Normal care provided by the primary health care team (i.e. general practitioners and health visitors) with no additional input (apart from assessment) from the research team.” |

## Appendix E: Details of Maternal and Child Outcomes by Type of Intervention Focus

**Table E.1: Maternal and child outcomes for mother-focused interventions**

| **S. No.** | **Study** | **Intervention** | **Maternal PPD Outcomes ^a^** |
| --- | --- | --- | --- |
|  | **MOTHER-FOCUSED: PSYCHOLOGICAL** | | |
|  | Quasi Experimental **Huynh-Nhu Le (2021)** | CBT | 1. **No significant reduction on PPD scores** PDSS-SF scores across groups at any time point; 2. **Significant reduction in PPD scores** for (a) completers, from T1 to T2, t(35) = −3.67, p =.001, and from T1 to T3, t(34) = −4.66, p =.00, with a non-significant decrease from T2 to T3; 3. **Non-significant reduction in PPD** scores for the non-completers; 4. **Significant reduction in PPD scores** for zero-class participants from T1 to T3, t(28) = −3.47, p =.002, and from T2 to T3, t(28) = −2.57, p =.016, with no significant change from T1 to T2. 5. **Significant reduction in** PPD Scores from T1 to T3 for all participants, F(2, 78) = 13.53, p =.00. |
|  | RCT  **Jannati (2020)** | CBT | 1. **Significant reduction in PPD scores** for both intervention and control groups   **Mean** EPDS scores before intervention: I = 17.42 ± 2.8 (range: 13–23); C = 17.39 ± 2.2 (range: 9–21), (p < 0.001)  **Mean** EPDS scores after intervention: I = 8.18 ± 1.5 (range: 6–11); C = 5.05 ± 2.9 (range: 9–21), (p < 0.001)  **Mean difference:** I = 9.24 units (p < 0.001); C= 2.34 units (p < 0.001).   1. **Significant difference in PPD scores** between intervention and control groups: 6.87 units; I = 8.18, SD = 1.5; C = 15.05, SD = 2.9 (p < 0.001). |
|  | Cluster RCT  **Ngai (2019)** | CBT | 1. **Significant difference in PPD scores** (measured in terms of low risk of PPD on EPDS) indicating group-by-time interaction effects on risk of PND (F = 3.69, P = 0.001).  - At 6 weeks postpartum, difference = 17.8% (95% CI 3.6–32.0), P = 0.01  1. **Non-significant difference in PPD scores** at 6 and 12 months |
|  | Cluster RCT  **Gureje (2019)** | PST | **(a) Non-significant reduction** in PPD scores **(**remission of PPD) at 6 months postpartum  Adjusted odds ratio = 1.3; 95% CI (0.8, 2.0); P=0.343  **(b)** **Significant reduction** in PPD scores for severe PPD  Interaction odds ratio = 2.29; 95% CI 1.01, 5.20; P=0.047  **[Child Outcomes**:  **(a)** **Non–significant improvements** in child growth and development;  **(b)** **Significant increase** in exclusive breastfeeding  19% v. 10%; odds ratio 2.17; 95% CI 1.27, 3.73; P=0.005**]** |
|  | RCT  **Lund (2019)** | Task-sharing multicomponent psychological | **(a) Non-significant reduction** in PPD scores at 3 and 12 months postpartum  3 months postpartum: RR=1.16; 95% CI 0.94, 1.43; p=0.153  12 months postpartum: RR=1.26; 95% CI 0.99, 1.60; p=0.057  **(b) Non-significant improvement** in recovery at 12 months postpartum  12 months postpartum: RR=1.49; 95% CI 0.92, 2.40; p=0.102  **[Child Outcomes**:  **(a)** **Non–significant improvements** in child growth  Weight: RR=1.03; 95% CI 0.94, 1.12; p=0.589  Height: RR=1.00; 95% CI 0.98, 1.04; p=0.619  Head circumference: RR=1.03; 95% CI 0.99, 1.06; p=0.163  **(b)** **Non-significant reduction** in diarrhoeal episodes or respiratory infections  Diarrhoea: RR=1.37, 95% CI 0.91, 2.06; p=0.126  Difficulty breathing: RR=0.89; 95% CI 0.63, 1.25; p=0.491  Cough: RR=1.12, 95% CI 0.90, 1.39; p=0.315  Hospital admission for difficult breathing: RR=0.64 95% CI 0.29, 1.43; p=0.276  **(c)** **Non-significant increase** in the likelihood of complete immunization  RR=1.02, 95% CI 0.94, 1.10; p=0.664**]** |
|  | RCT  **Fuhr (2019)** | BA | **(a) Non-significant reduction** in PPD scores at 6 months  Mean PHQ-9 score I = 3·47 [SD 4·49] vs. C = 4·48 [5·11]  SMD –0·18, 95% CI –0·43 to 0·07; p=0·16  **(b) Significant decrease** in PPD prevalence (or higher prevalence of remission) at 6 months   - Proportion of women: I = 89 [73%] vs. C = 77 [60%] - Prevalence ratio (PR)=1·21, 95% CI 1·01 to 1·45; p=0·04   **(c) Significant reduction** in PPD scores at 3 months   - SMD –0·34, 95% CI –0·59 to –0·09; p=0·01   **(d) Non-significant decrease** in PPD prevalence (or prevalence of remission) at 3 months  p=0·08  **(e)** **Significant decrease** in overall prevalence of PPD (improved recovery, i.e., PHQ9 score<5 at 3 and 6 months postpartum)  PR=1·35, 95% CI 1·03 to 1·78; p=0·03). |
|  | RCT  **Sikander (2019)** | BA | **(a) Non-significant reduction** in PPD scores at 6 months  SMD=-0·13, 95% CI -0·31 to 0·06, p=0·07; 49% vs. 45%;  **(b) Non-significant decrease** in PPD prevalence (or prevalence of remission) at 6 months  PR=1·12, 95% CI 0·95 to 1·29, p=0.14   - Repeated measures analyses over the 6 months post childbirth: SMD=-0·22, 95%CI -0·35 to -0·09, p=<0·001) and PR=1·15 95% CI 1·02 to 1·28, p=0·02   **(c) Significant reduction** in PPD scores at 3 months  SMD=-0·30, 95%CI -0·48 to -0·11, p<0·001  **(d) Significant decrease** in PPD prevalence (or prevalence of remission) at 3 months  PR=1·18, 95%CI 1·06 to 1·29, p=0·002)  **(e)** **Significant decrease** in overall prevalence of PPD (recovery)  Improved recovery (PR=1·36, 95%CI 1·09 to 1·63, p=0.002). |
|  | RCT **Dimidjan (2017)** | BA | (a) **Significant reduction** in PPD scores averaged across follow-up time points  *F*(1, 152)=4.39; d=0.34 (p=.04)  (b) **Clinically significant improvement** for BA at 3-months postpartum  Odds ratio [OR]=2.05 (p = 0.004; CI: 1.06 –3.99) |
|  | RCT **Forsell (2017)** | CBT | (a) **Significant reduction** in in PPD scores post-treatment  Effect size Hedges’ g = 1.21 (p<0.001) (b) **Significant improvement** in PPD symptoms post-treatment  RR = 0.36 (p = 0.004, 95% CI = 0.16–0.82)  (c) **Significant decrease** in PPD prevalence post treatment  RR = 0.42 (p = 0.002, 95% CI = 0.23–0.77) |
|  | RCT **Pugh (2016)** | CBT | (a) **Significant reduction** in PPD scores at 10 weeks follow-up and at 4 weeks follow-up post treatment completion   - F (1,20.99) = 16.23 (p = .001); a condition by time interaction, F (1, 11.82) = 5.15 (p = .02). - **Statistically significant improvement at T3** [t(14) = 4.13, d = 1.10 (p <.01)] |
|  | RCT **Milgrom (2016)** | CBT | (a) **Significant reduction** in severity of PPD symptoms at 12 weeks follow-up Yates corrected χ2 1=10.3, F 1,40 = 7.4, d = 0.83 (P= .01; 95% CI = (0.20, 1.45)  (b) **Significant reduction** in PPD prevalence at 12 weeks follow-up |
|  | RCT **Jesse (2015)** | CBT | (a) **Non-significant reduction in** PPD scores  (b) **Significant reduction** in PPD scores in women at low-moderate risk  T1 to T2 (4.92 vs. 0.59, Pv=v.018) and T1 to T3 (5.67 vs. 1.51, P=.04).  (c) **Significant reduction** in PPD scores for at-high-risk African-American  T1 to T2 (5.59 vs. 2.18, P=.02) and from T1 to T3 (6.32 vs. 3.14, P= .04). |
|  | Quasi-experimental  **Ashtiani (2015)** | CBT | (a) **Significant reduction in** average PPD scores at 2 weeks postpartum Pre-test (mean ± SD) = 21.17±10.92; Post-test (mean ± SD) = 14.86±5.95; P(between groups) = 0.001 |
|  | Quasi Experimental clinical trial **Dimidjan (2015)** | MBCT (Mindfulness based cognitive therapy) | **(a) Significant reduction** in PPD scores with sustained decrease during the intervention  F(1,49)=8.55, d=0.84 (p=0.0037)  **Sustained decrease**  t(48)=2.48, d=0.71 (p=0.013) |
|  | RCT **Tandon (2014)** | CBT | (a) **Significant reduction in** PPD scores at 1 week, 3 months and 6 months post intervention  Unstandardized coefficient = −6.07; z = −2.96; d = −0.64 (P < 0.01)  At 3 months unstandardized coefficient = −5.91; z = −2.87; d = −0.62 (P < 0.01)  At 6 months unstandardized coefficient = −6.94; z = −3.35; d = −0.73 (P < 0.001)  (b) **Non-significantly lower** PPD incidence at 6 months post-intervention  11/34 (32.4%) Controls and 6/41 (14.6%) intervention group  (χ2 = 3.33, P = .07, effect size (φ) = .21) |
|  | RCT **Jiang (2014)** | CBT | (a) S**ignificant reduction** in PPD scores at 6 months postpartum  12.84±3.02 to end stage 3.05±2.93  t=13.059, (P<0.001) |
|  | Quasi-experimental study **Hou (2014)** | CBT + Systemic Family Therapy | (a) **Significant reduction** in PPD scores post intervention and at 6, 12, 18 and 24 months follow-up after intervention  t = -5.40 (p<0.001) (b) **Significantly higher reduction at last stage** (24 months)  t = 6.49 (p<0.01) |
|  | RCT **Pinheiro (2013)** | CBT and RCT | (a) **Significant reduction** for **both CBT (p = 0.05) and RCT (p < 0.001)**: No significant between group difference  (a) **Non-significant reduction** in PPD scores at 12 months after intervention  (b) **Non-significant reduction** in PPD prevalence (p = 0.139) |
|  | RCT **Ammerman (2013)** | CBT | (a) **Non-significant** reduction in PPD scores  (b) **Significant reduction** in PPD prevalence post-treatment and at 3 months follow-up after treatment  (29.3% vs. 69.8%, OR = 0.18) and follow-up (20.5% vs. 52.6%, OR = 0.23) p<.01 |
|  | RCT **O'Mahen (2013)** | BA | (a) **Significant reduction** in in PPD scores for non-depressed participants at 15 weeks: favoring Postnatal-iBA group (n=115/181; 63%) compared to TAU (n=71/162; 43.8%, p<.001); OR of 2.16 (95% CI 1.38, 3.37).  (b) **Significant reduction** in PPD scores in individuals with mild–moderate depressive symptoms (χ2 (1) = 8.67, p<.01).  (c) **Significant reduction** in PPD scores in individuals with more severe depressive symptoms (χ2 (1) = 3.98, p=.05). **Phase-II** (d) **Significant reduction in** PPD prevalence  Cohen’s d effect sizes, EPDS (−0.87, 95% CI −0.42 to −1.32), Cohen’s d effect sizes, GAD-7 (−0.59, 95% CI −1.11 to −0.07). |
|  | RCT **Milgrom (2011)** | CBT | (a) **Non-significant reduction** in PPD scores across different treatment groups  F= 1.051, do = 2,45 (p = .358).  (b) **Significant increase** in PPD prevalence in GP management group  Χ2, df = 2, (p = .028) |
|  | RCT **Le (2011)** | CBT | (a) **Significant reduction in** PPD scores at time point 2 (late pregnancy)  Cohen’s d= - 0.28 (p=.03).  (b) **Significantly lower incidence** of moderate depression at time point 2 (late pregnancy) |
|  | Cluster RCT **Brugha (2011)** | CBT or person centred | (a) **Significant reduction** in PPD scores at 6 months postpartum  OR at 6 months = 0.71 [p=0.031, 95% CI=0.53–0.97] |
|  | RCT, **Austin (2008)** | CBT | (a) **Non-significant reduction** in PPD scores at 6 weeks post-intervention, 2 months postpartum and 4 months postpartum (means 6.88 - 8.16)  (b) **Non-significant reduction** in PPD prevalence |
|  | **MOTHER-FOCUSED: PSYCHOSOCIAL** | | |
|  | RCT **Lara (2010)** | Psychoeducational, non-directive counseling, supportive interactions via group sessions | (a) **Non-significant reduction** in PPD scores  (b) **Significantly lower** cumulative incidence of major PPD  Intervention group incidence = 10.7% (6/56); Control group incidence = 25% ((15/60); X2(1) =5.356 (p<0.05) |
|  | RCT  **Dennis (2009)** | Supportive interactions via peer support | **(a) Significant reduction** in PPD scores at 12 weeks postpartum OR: 2.1, 95% CI: 1.38 to 3.20  **χ**2**=**12.5, P<0.001; relative risk reduction 0.46, 0.24 to 0.62)  **(b) Non-significant reduction** in PPD scores at 24 weeks postpartum  **χ**2**=**2.53, P**=**0.11, I = 11% (33/289) vs. C = 14% (43/311) participants scored>12 on EPDS (OR 1.22, 0.75 to 1.98) |
|  | RCT **Ho (2009)** | Psychoeducational strategies | (a) **Non-significant reduction** in PPD scores at 6 weeks and at 3 months postpartum  6 weeks: (Χ2 = 1.90, df = 1, p = 0.17; t = 5.65, 95% CI=1.50 - 3.13)  3 months: (11% I versus 16% C; Χ2 = 1.02, d.f. = 1, p = 0.31; 95% CI, *p* = 4.46, 0.94 to 2.44 <0.001) |
|  | **MOTHER-FOCUSED: MIXED** | | |
|  | RCT **Gao (2014)** | IPT + Psychoeducation | (a) **Significant reduction** in PPD scores at 6 weeks postpartum  Mean [SD]: I = 7.61 [3.43], C = 8.96 [4.55]; t = 2.24; (p = 0.026) |
|  | RCT **Kozinsky (2012)** | Group therapy + IPT + CBT elements + psycho education | (a) **Significant reduction** in PPD prevalence 6 weeks postpartum  (OR = 0.69) - absolute risk reduction of 18% for depression and 0.5% for no depression at recruitment. |
|  | RCT **Gao (2011)** | IPT + Routine antenatal education | (a) **Significant reduction** in PPD scores at 3 months postpartum  t = 2.39 (p=0.018) |

^a^ *The PPD outcomes include: (1) PPD diagnosis made by clinicians using diagnostic tools such as SCID-IV or MINI (2) Severity of PPD symptoms, i.e., having some of the features of depression but not the full list to qualify for a diagnosis.*

**Table E.2**

*Maternal and child outcomes for mother-child focused interventions*

| **S. No.** | **Study** | **Intervention** | **Maternal PPD Outcomes ^a^** | **Child Outcomes** |
| --- | --- | --- | --- | --- |
|  | **MOTHER-CHILD FOCUSED: PSYCHOLOGICAL** | | | |
|  | Cluster RCT **Rahman (2008)** | CBT | (a) **Significant reduction** in PPD prevalence at 6 and 12 months postpartum  6 months postpartum (aOR: 0.22; P < 0.0001);  12 months postpartum (aOR 0.23; P < 0.0001; 95% CI: 0.15–0.36;) | (a) **Non-significant** reduction in infant stunting or malnutrition (b) **Significant reduction** in diarrheal episodes at 12 months of child’s age (aOR: 0.6; P = 0.04; 95% CI: 0.39–0.98;),  (c) **Significant increase in** the likelihood of complete immunization of infants at 12 months of child’s age  (aOR: 2.5; P = 0.001; 95% CI: 1.47–4.72;). |
|  | **MOTHER-CHILD FOCUSED: PSYCHOSOCIAL** | | | |
|  | Cluster RCT **Tripathy (2010)** | Supportive interactions via participatory group meetings | (a) **Non-significant reduction** in PPD scores  (b) **Significantly lower** incidence of moderate depression in year 3 of the study aOR 0.43 (95% CI: 0.23–0.80) | (a) **Significant reduction** in NMR  32% lower in intervention (aOR: 0.68; 95% CI: 0.59–0.78); 45% lower in years 2 and 3 (aOR: 0.55; 95% CI: 0.46–0.66) |
|  | RCT **Cooper (2009)** | Psychoeducational strategies + counseling support via home visits | (a) **Significant reduction in** PPD scores at 6 months postpartum only  z=2.05 (P=0.04) **(b) Quality of mother-infant interactions:**   - **Significant increase** in mother’s sensitivity at 6 months postpartum: mean difference=0.77, SD 0.37, t=2.10, d=0.2 (P<0.05,) - **Significant difference** in mother’s sensitivity at 12 months postpartum: mean difference=0.42, SD=0.18, t=−2.04, d=0.26 (P<0.05) - **Significant reduction** in intrusiveness of mothers at 6 months**:** mean difference=0.68, SD=0.36), t=2.28, d=0.26 (P<0.05) - **Significant reduction** in intrusiveness of mothers at 12 months: mean difference=−1.76, SD=0.86), t=2.28, d=0.24 (P<0.05) | (a) **Significant increase in** more securely attached infants at 18 months of child’s age: 116/156 (74%) v 102/162 (63%); Wald=4.74, odds ratio=1.70, (P < 0.029) |
|  | Cluster RCT **Henningham (2005)** | Psychoeducational strategies + supportive interactions | (a) **Significant reduction** in PPD scores   - **Largest reduction** for 40–50 home visits: β = −1.84 (95% CI: –2.97 to –0.72); - **Lesser reduction** for 25–39 home visits: β = −1.06 (95% CI: –2.02 to –0.11) - N**on-significant reduction** for 0–24 home visits: β = −0.09 (95% CI: –1.11 to 1.13) | (a) **Non-significant** for child development  (b) **Significant correlation** between final PPD and DQ scores in boys only  (P < 0.05) (b)  (c) Child Growth: Not reported |
|  | **MOTHER-CHILD FOCUSED: MIXED** | | | |
|  | RCT  **Stein (2018)** | CBT + VFT VS. CBT + PMR | **(a) Non-significant reduction** in PPD scores   - 1-year and 2-year time-points: improvement in both treatment groups, significant changes from baseline within each group (p<0·0001) - No significant difference at 1 year (p=0·68) - At 2 years, PMR group scored significantly lower than the VFT group | (a) **Non-significant difference** for child development and behavioural outcomes   - Cognitive development, adjusted difference –1·01 [95% CI –5·11 to 3·09], p=0·63; - Language development, 1·33 [–4·16 to 6·82], p=0·63; - Behaviour problems, –1·77 [–4·39 to 0·85], p=0·19; - Attachment security, 0·02 [–0·06 to 0·10], p=0·58), with |
|  | RCT **Husain (2017)** | CBT + non-directive counseling + supportive interactions | (a) **Significant reduction** in PPD scores at 3 months  Adjusted mean difference [AMD] = -3.65 (95% CI -6.14 to -1.15, p<0.004);  Effect sustained at 6 months  AMD = -2.62 (95% CI -4.43 to -0.81, p<0.005). | (a) **Non-significant difference** in height and weight measure |
|  | RCT **Cooper & Murray (2003)** | CBT + Psychodynamic therapy + non-directive supportive counseling | (a) **Non-significant reduction** in PPD scores for all three treatments at 4.5 months postpartum only  (b) **Significant** **reduction** in PPD scores in psychodynamic therapy group only  (c) **Non-significant reduction** in PPD prevalence at 5 years postpartum | (a) **Non-significant improvement** in management of infant behaviour, mother-infant attachment and infant cognitive development at 5 years  (b) **Significant reduction** in emotional and behavioural problems in infants for non-directive counseling at 18 months postpartum only |

^a^ The PPD outcomes include: (1) PPD diagnosis made by clinicians using diagnostic tools such as SCID-IV or MINI (2) Severity of PPD symptoms, i.e., having some of the features of depression but not the full list to qualify for a diagnosis.
